# YWHAZ-mediated metabolic reprogramming via HIF1A/LDHA signaling promotes pulmonary arterial remodelling

**DOI:** 10.1038/s41420-026-03121-y

**Published:** 2026-05-05

**Authors:** Zhong-Yuan Meng, Chuang-Hong Lu, Juan Liao, Sen-Hu Tang, Jing Li, Xiao-Li Ma, Yue Qin, Chao-Yong Zhang, Yao-Shi Hu, De-Xin Chen, Xing Chen, Yan Deng, Feng Huang, Zhi-Yu Zeng

**Affiliations:** 1 Department of Cardiology, The First Affiliated Hospital of Guangxi Medical University, No.6 Shuangyong Road, Nanning, Guangxi 530021 China; 2 Department of Cardiology, Affiliated Hospital of Youjiang Medical University for Nationalities, No.18 Zhongshan 2nd Road, Youjiang District, Baise City, Guangxi 533000 China; 3 Guangxi Key Laboratory of Precision Medicine in Cardio-cerebrovascular Diseases Control and Prevention, Guangxi Clinical Research Center for Cardio-cerebrovascular Diseases, No.6 Shuangyong Road, Nanning, Guangxi 530021 China; 4 The Key Laboratory for High Incidence Prevention and Treatment in Guangxi Guixi Area, Youjiang Medical University for Nationalities, Baise, 533000 Guangxi China; 5 Ultrasound Department, The First Affiliated Hospital of Guangxi Medical University, No.6 Shuangyong Road, Nanning, Guangxi 530021 China; 6 Department of Cardiology, Liuzhou People’s Hospital, Guangxi, Zhuang Autonomous Region, Wenchang Road 8, Liuzhou, 545000 China

**Keywords:** Cell growth, Molecular biology

## Abstract

Hypoxia-related pulmonary arterial hypertension (PAH) remains poorly managed by current therapies. Metabolic dysregulation, particularly glycolysis, plays a key role in PAH pathogenesis. This study investigated YWHAZ’s role in PAH using hypoxia-induced pulmonary arterial endothelial cells (PAECs) and a hypoxia/SU5416-induced PAH rat model. Silencing YWHAZ inhibited PAEC proliferation, migration, and glycolysis, while improving right ventricular function and reducing pulmonary vascular remodeling. Mechanistically, YWHAZ stabilized HIF-1α, which transcriptionally activated LDHA, a critical glycolytic enzyme. HIF-1α agonist treatment reversed YWHAZ silencing effects, confirming the YWHAZ/HIF-1α/LDHA axis. These findings highlight YWHAZ as a potential therapeutic target for metabolic intervention in PAH.

## Introduction

Pulmonary arterial hypertension (PAH) is a pathophysiological syndrome caused by diverse etiologies and pathogenic mechanisms, primarily characterized by a significant increase in pulmonary vascular resistance, elevated pulmonary arterial pressure, pulmonary vascular remodeling, and increased right ventricular afterload [1, 2]. These pathological changes eventually lead to right ventricular hypertrophy and further development of right heart failure. Epidemiological data indicate that at least 1% of the global population is affected by PAH [1, 3]. Despite significant advances in the treatment of PAH, it remains challenging [4]. The 5-year mortality rate of patients with PAH remains around 50% [5]. Current clinical therapies primarily rely on vasodilators, which can improve pulmonary vasodilation but have limited efficacy in reversing pulmonary vascular remodeling [5–7]. Hypoxia-related PAH is classified as Group 3 PAH, for which most approved PAH therapies exhibit limited effectiveness and may even have adverse effects in some cases [6, 8]. Therefore, exploring novel mechanisms and therapeutic strategies targeting pulmonary vascular remodeling is of critical importance.

Metabolic alterations, particularly aerobic glycolysis (i.e., the Warburg effect), are increasingly recognized as one of the important pathogenic mechanisms in the development of PAH [9, 10]. A previous study found significantly increased glucose uptake in the lungs of PAH patients compared to healthy controls, confirming enhanced glycolytic activity in PAH [11]. Another study reported marked metabolic dysregulation in blood outgrowth endothelial cells from PAH patients, characterized by a shift from oxidative phosphorylation to aerobic glycolysis [12]. Importantly, this study demonstrated that overexpression of miR-124 or silencing of PTPB1 could reverse the abnormal glycolysis in these cells and restore their normal proliferative capacity [12].

Tryptophan 5-monooxygenase-activating protein zeta (YWHAZ) is a key member of the 14-3-3 family of proteins, which is widely involved in a variety of signal transduction processes [13]. Studies have shown that YWHAZ plays an important role in various types of tumors, promoting tumor cell proliferation [14, 15], apoptosis resistance [16], migration [17], and angiogenesis [18]. Recent evidence suggests that YWHAZ can regulate the occurrence of glycolysis, such as YWHAZ can over-regulate glycolysis to promote ovarian cancer metastasis [19]. Quercetin inhibits glycolysis and proliferation in oral squamous cell carcinoma through the G3BP1/YWHAZ axis [20]. In addition, it has also been reported in the literature that YWHAZ is able to promote the proliferation and invasion of tumor cells by enhancing the glycolysis level of lung cancer cells and pancreatic cancer cells [21, 22]. Notably, elevated fasting insulin levels were observed in YWHAZ knockout mice, while maintaining normal β-cell responsiveness to glucose and significant improvement in glucose tolerance [23]. Collectively, these findings highlight YWHAZ as a crucial regulator of glycolysis. However, its role in the development of PAH remains unexplored.

In this study, we investigated the role of YWHAZ-mediated glycolysis in pulmonary artery endothelial cells (PAECs) proliferation and pulmonary vascular remodeling using hypoxia-induced PAH rat models and primary PAEC cultures. Our findings further reveal that YWHAZ may promote glycolysis by modulating the HIF-1α signaling pathway, thereby driving abnormal PAEC proliferation and vascular remodeling. This study provides novel insights into the impact of YWHAZ in PAH progression and suggests a potential therapeutic strategy.

## Result

### YWHAZ expression was significantly elevated in PAH

**Fig. 1:**
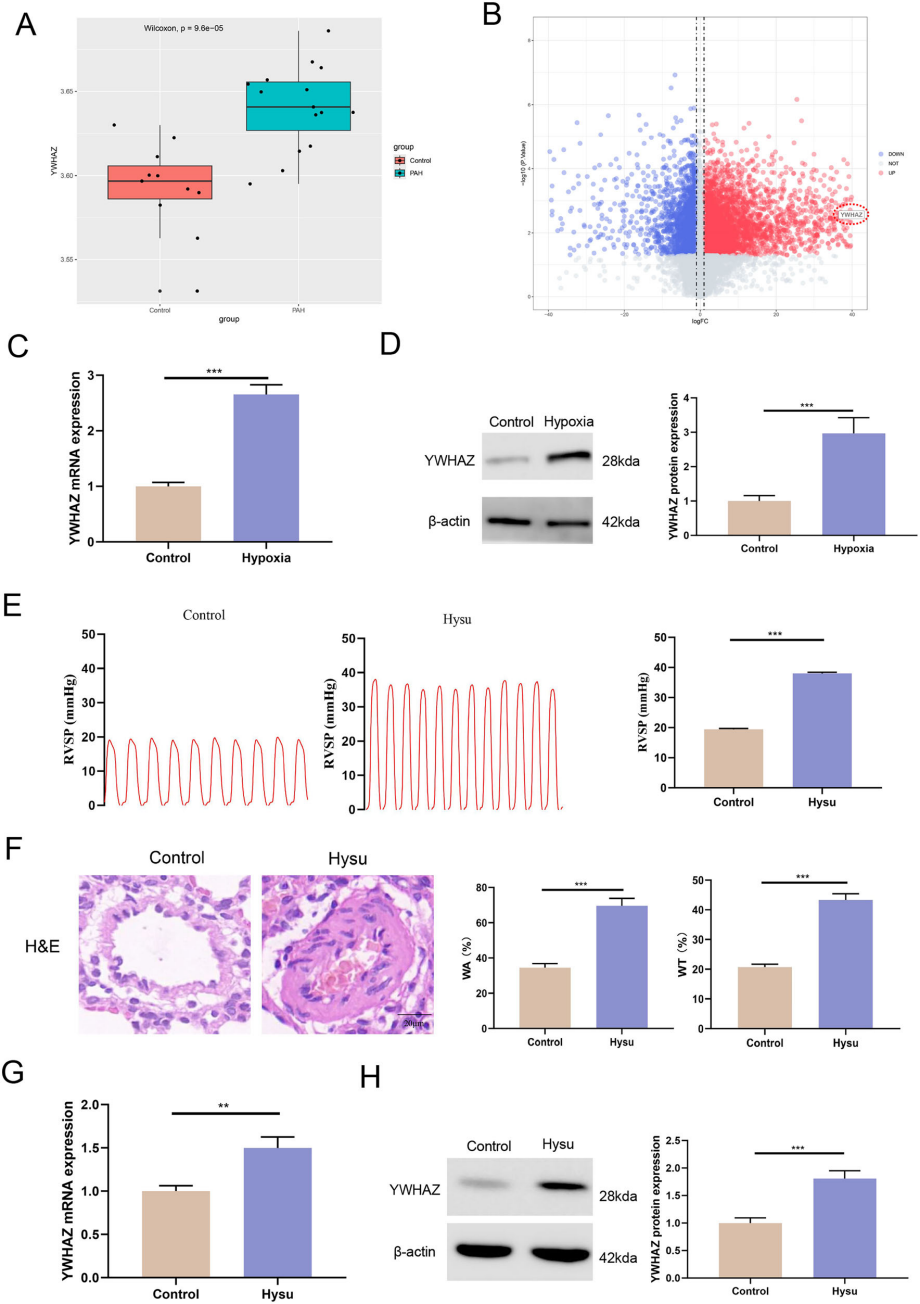
YWHAZ expression was significantly elevated in PAH. **A** YWHAZ expression was significantly elevated in PAH patients in the GSE113439 dataset. **B** In transcriptome analysis, YWHAZ expression was significantly elevated in hypoxic PAECs. qPCR (**C**) and Western blot (**D**) revealed that YWHAZ expression was significantly elevated in hypoxic PAECs (n = 4). **E** RVSP was measured in rats under normoxic and hypoxic conditions (n = 6). **F** H&E stained were used to measure the internal diameter and thickness of pulmonary artery walls (n = 6). qPCR (**G**) and Western blotting (**H**) were employed to determine relative YWHAZ mRNA and protein expression levels in rat lung tissues (n = 5-6). All data were presented as the mean ± SEM.*P < 0.05, **P < 0.01 and ***P < 0.001.

YWHAZ expression was significantly elevated in PAH patients In the GSE113439 (Fig. 1A). Transcriptome analysis showed that YWHAZ expression was also significantly increased in hypoxic PAECs (Fig. 1B). Subsequent qPCR and western blot further confirmed that YWHAZ expression was significantly elevated in hypoxic PAECs (Fig. 1C, D). Next, we constructed a hypoxia-induced PAH animal model, which showed that hypoxia-induced PAH rats had elevated right ventricular systolic pressure (RVSP) (Fig. 1E), and decreased internal diameter and increased thickness of pulmonary artery walls (Fig. 1F). Similarly, qPCR and WB results showed that YWHAZ expression was significantly elevated in hypoxia-induced PAH rats (Fig. 1G, H).

### Silencing YWHAZ inhibited hypoxia-induced PAECs proliferation, migration, and glycolysis

**Fig. 2:**
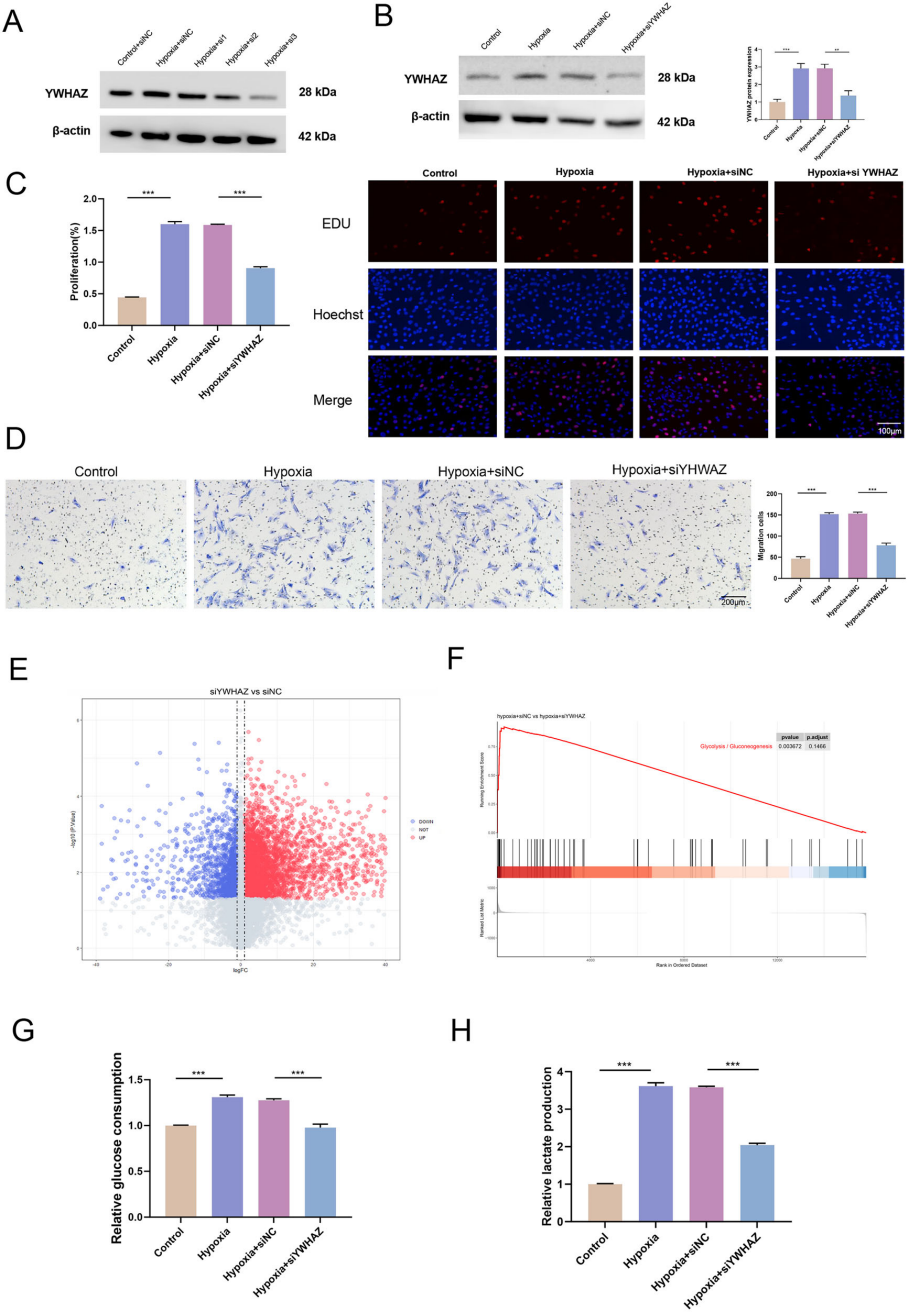
Silencing of YWHAZ inhibits PAECs proliferation, migration and glycolysis levels. **A**, **B** siYWHAZ effectively reduced the expression of YWHAZ (n = 4). **C** Silencing YWHAZ can inhibit hypoxia-induced PAECs proliferation (n = 4). **D** Silencing YWHAZ attenuated hypoxia-induced PAECs migration (n = 4). **E** Heatmap representation of DEGs from RNA-seq. **F** Visualization of GSEA enrichment analysis. **G** Measurement of glucose uptake by PAECs in each group (n = 4). **H** Measurement of lactate production in PAECs of each group (n = 4). All data were presented as the mean ± SEM.*P < 0.05, **P < 0.01 and ***P < 0.001.

To investigate the biological role of YWHAZ, we silenced the expression of YWHAZ by transfecting primary PAECs with a small interfering RNA (siRNA) of YWHAZ. The siRNA-3 showed the highest silencing efficiency, and was selected for subsequent experiments (Fig. 2A). Western blot assay showed that the inhibition rate of siRNA on YWHAZ was more than 50% (Fig. 2B). EdU assays revealed that hypoxic PAECs exhibited significantly enhanced proliferation compared to normoxic controls, whereas YWHAZ silencing markedly attenuated hypoxia-induced proliferation (Fig. 2C). Similarly, transwell assays demonstrated that YWHAZ knockdown significantly inhibited the migratory capacity of hypoxic PAECs (Fig. 2D). To further explore whether other phenotypic changes would be affected after intervention of YWHAZ, we performed transcriptome sequencing analysis. RNA-seq identified 4822 differentially expressed genes (DEGs), including 3,371 upregulated and 1,451 downregulated genes (Fig. 2E). Gene Set Enrichment Analysis (GSEA) of these DEGs suggested potential regulation of glycolytic pathways (Fig. 2F). Subsequent Warburg effect-related analyses showed that YWHAZ knockdown significantly reduced glucose uptake and lactate production compared to the siNC group (Fig. 2G, H).

### Silencing YWHAZ inhibited HIF1α expression

**Fig. 3:**
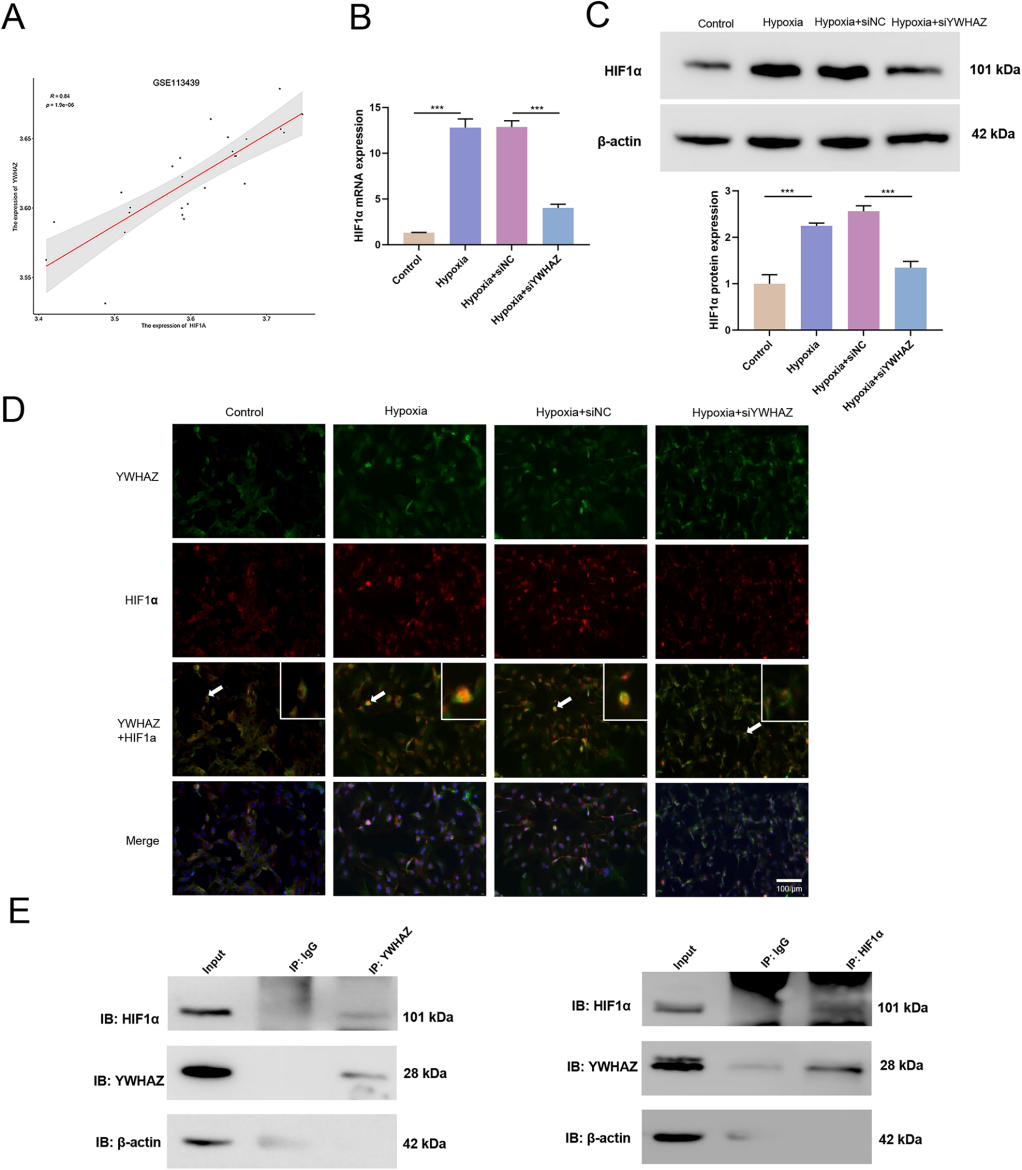
YWHAZ regulated HIF1α expression. **A** Correlation analysis between YWHAZ and HIF1α expression level. RT-qPCR (**B**) and Western blot (**C**) were used to detect the expression level of HIF1α in PAECs in each group (n = 4). **D** YWHAZ regulates the nuclear translocation of HIF1α under hypoxic conditions. **E** YWHAZ interacts with HIF1α. All data were presented as the mean ± SEM.*P < 0.05, **P < 0.01 and ***P < 0.001.

Spearman correlation analysis revealed a significant positive correlation between YWHAZ and HIF1α expression levels (r = 0.84, *P* < 0.001; Fig. 3A). qPCR and WB further indicated that silencing of YWHAZ inhibited the expression of HIF1α (Fig. 3B and C). In addition, we observed an intriguing phenomenon. Under hypoxic conditions, YWHAZ was co-translocated from the cytoplasm to the nucleus with HIF1α. This hypoxia-induced nuclear translocation of HIF1α appeared to be attenuated upon YWHAZ knockdown (Fig. 3D), suggesting that YWHAZ may play a crucial role in regulating HIF1α nuclear translocation during hypoxia. Co-immunoprecipitation experiments demonstrated a direct protein interaction between YWHAZ and HIF1α (Fig. 3E). To investigate whether this interaction affects HIF-1α protein stability, we treated PAECs with cycloheximide (CHX), an inhibitor of de novo protein synthesis. The results showed that YWHAZ silencing significantly reduced HIF-1α protein stability (Supplementary Fig. 2A). Furthermore, YWHAZ knockdown increased the ubiquitination of HIF-1α (Supplementary Fig. 2B).

### DMOG partially reversed the siYWHAZ-mediated suppression of YWHAZ expression, glucose consumption, and lactate production

**Fig. 4:**
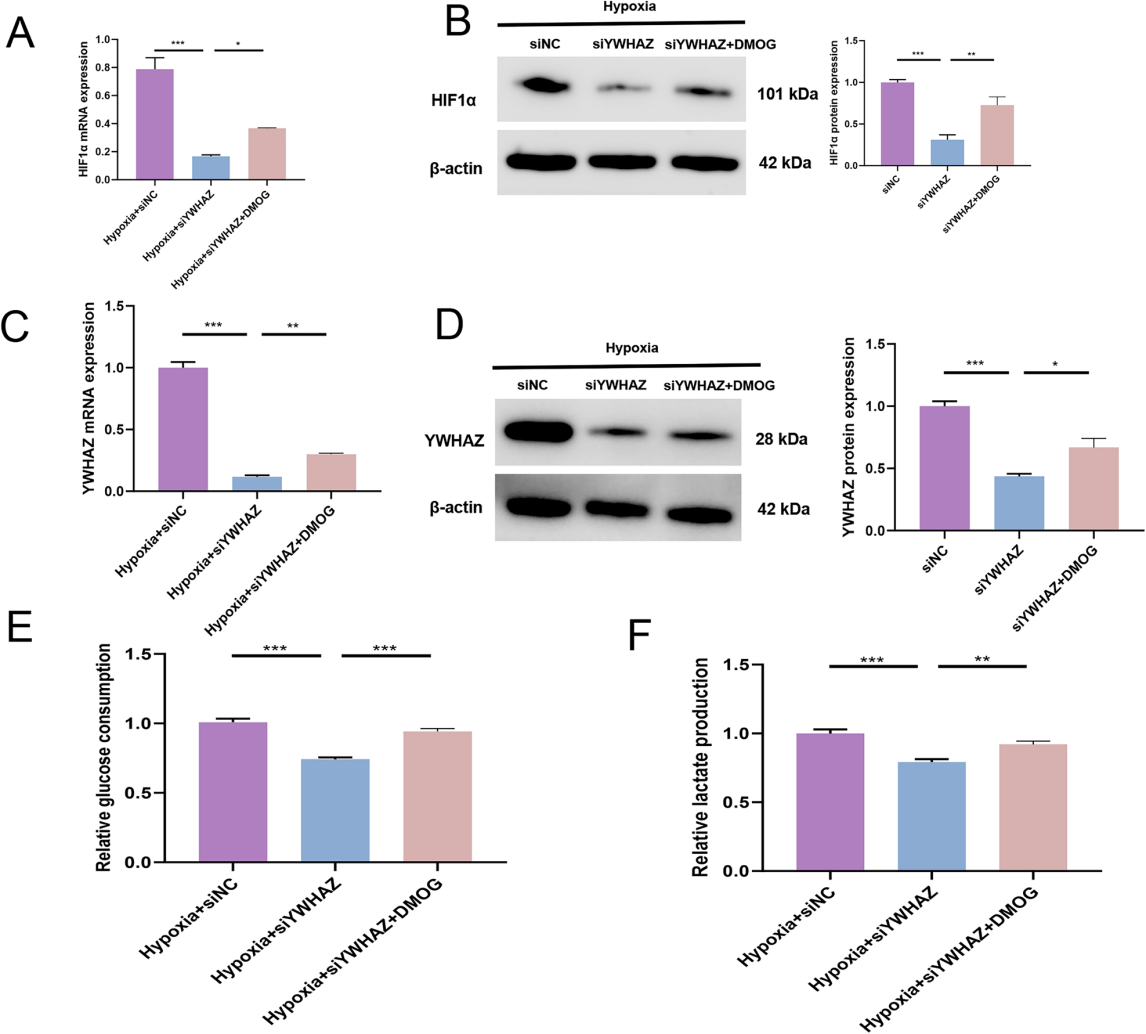
DMOG partially reversed siYWHAZ-mediated suppression of YWHAZ expression, glucose consumption, and lactate production. qPCR (**A**) and Western blot (**B**) were used to detect the expression level of HIF1α in different intervention groups (n = 4). qPCR (**C**) and Western blot (**D**) were used to detect the expression level of YWHAZ in different intervention groups (n = 4). **E** Measurement of glucose uptake by PAECs in each group (n = 4). **F** Measurement of lactate production in PAECs of each group (n = 4). All data were presented as the mean ± SEM.*P < 0.05, **P < 0.01 and ***P < 0.001.

qPCR and western blot results showed that DMOG increased the expression level of HIF1α (Fig. 4A, B). Moreover, DMOG partially reversed siYWHAZ inhibition of YWHAZ expression (Fig. 4C, D). DMOG intervention partially reversed the inhibitory effect of silencing YWHAZ on glucose uptake, and lactate production (Fig. 4E, F). In addition, inhibition of LDHA (Oxamate, MCE, 565-73-1, China) yielded effects comparable to those observed upon YWHAZ silencing in pulmonary artery endothelial cells, including no significant differences in cell proliferation (Supplementary Fig. 3), migration (Supplementary Fig. 4), glucose uptake (Supplementary Fig. 5A), or lactate production (Supplementary Fig. 5B).

### YWHAZ mediated HIF1α-regulated LDHA expression

**Fig. 5:**
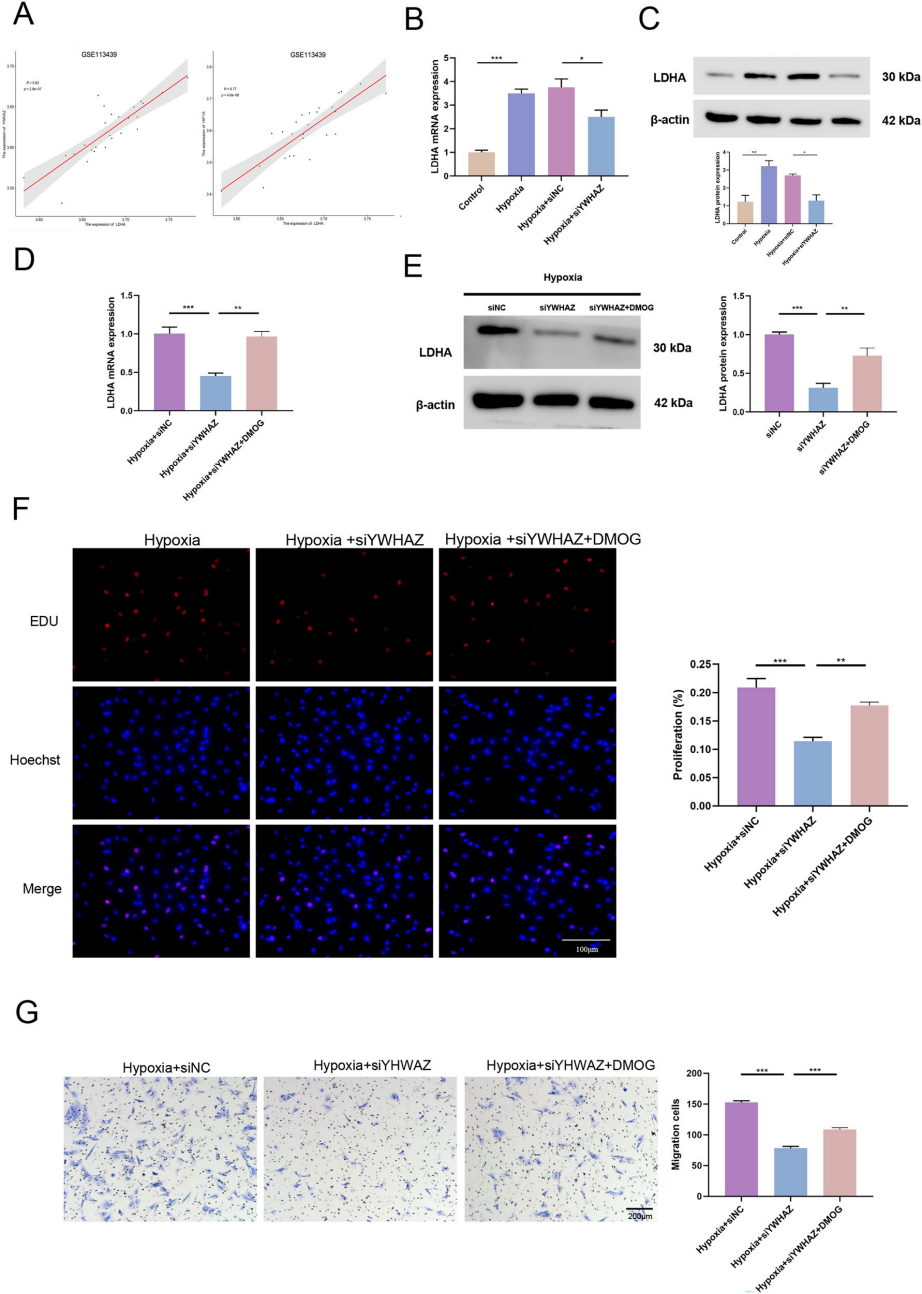
YWHAZ regulated LDHA expression, and DMOG partially reversed siYWHAZ-mediated suppression of LDHA expression, PAECs proliferation and migration. **A** Correlation analysis of LDHA expression with YWHAZ and HIF1α levels. RT-qPCR (**B**) and western blot (**C**) detected the expression level of HIF1α in different intervention groups (n = 4). **D**, **E** DMOG treatment partially rescued the siYWHAZ-induced suppression of LDHA expression (n = 4). DMOG partially reversed the effect of siYWHAZ on PAECs proliferation (**F**) and migration (**G**) (n = 4). All data were presented as the mean ± SEM.*P < 0.05, **P < 0.01 and ***P < 0.001.

The expression level of YWHAZ was significantly positively correlated with LDHA expression (r = 0.82, *P* < 0.001; Fig. 5A). Similarly, HIF1α expression was also positively correlated with LDHA levels (r = 0.77, *P* < 0.001). Silencing of YWHAZ significantly suppressed LDHA expression (Fig. 5B, C). Furthermore, the expression level of LDHA was partially reversed after DMOG intervention (Fig. 5D, E). DMOG also partially reversed the inhibitory effect of YWHAZ knockdown on hypoxia-induced PAECs proliferation and migration (Fig. 5F, G).

### Silencing YWHAZ improved RVSP, right heart function and pulmonary artery remodeling in PAH Rats

**Fig. 6:**
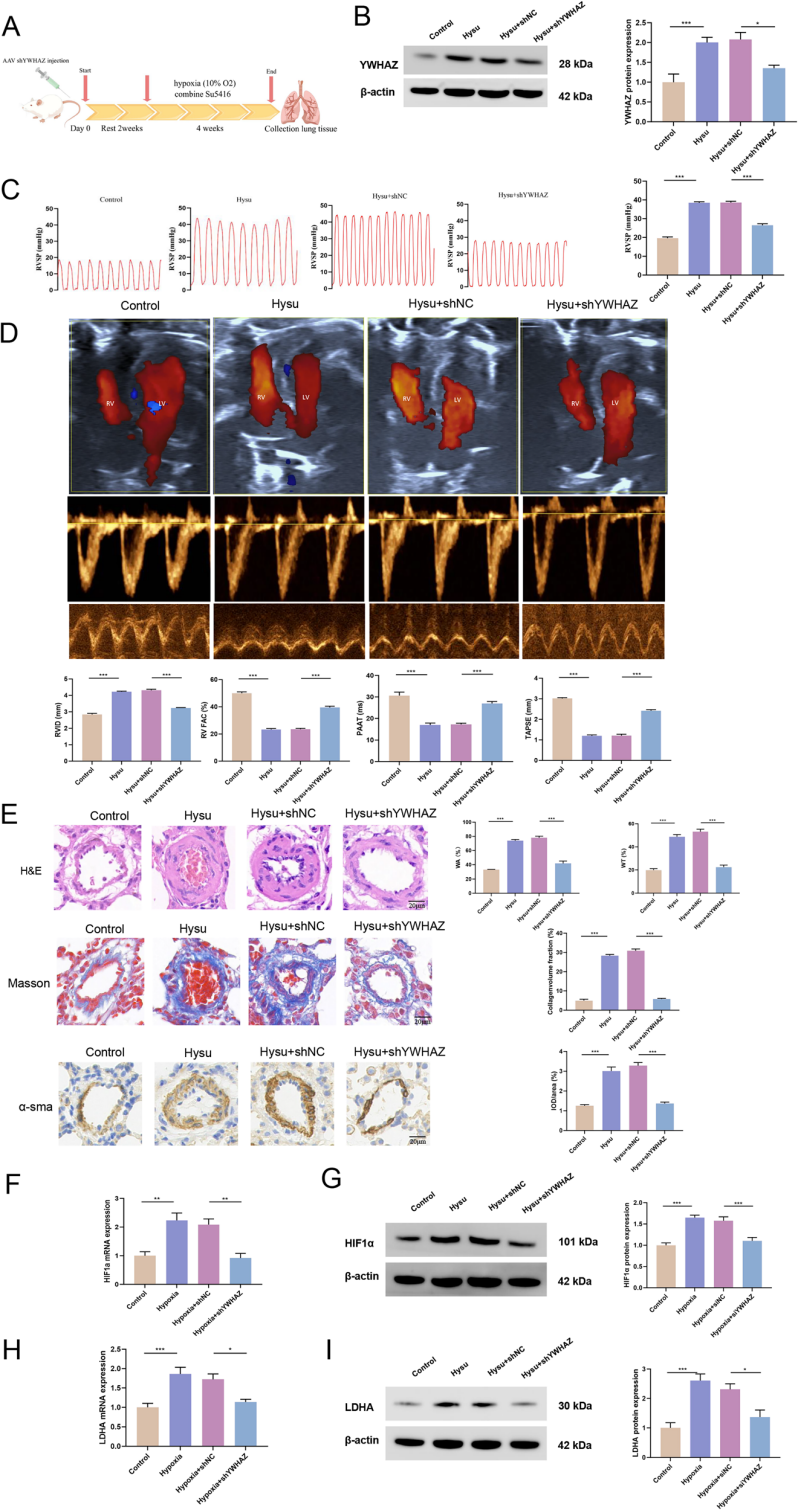
Silencing YWHAZ ameliorated RVSP, right heart function and pulmonary artery remodeling in PAH rats. **A** Schematic diagram of the experimental design for intervention in rats. **B** shYWHAZ inhibition effectively reduced YWHAZ expression in rat lung tissues (n = 6). **C** YWHAZ inhibition significantly reduced RVSP in PAH rats (n = 6). **D** YWHAZ inhibition was able to reduced RVID and improved RV FAC%, PAAT and TAPSE in PAH rats (n = 5). **E** YWHAZ inhibition improved pulmonary arterial remodeling, reduced collagen deposition, and decreased vascular muscularization in PAH rats (n = 5-6). RT-qPCR (**F**) and Western blot (**G**) were used to detect the expression level of HIF1α in different intervention groups (n = 6). RT-qPCR (**H**) and Western blot (**I**) were used to detect the expression level of LDHA in different intervention groups (n = 6). All data were presented as the mean ± SEM.*P < 0.05, **P < 0.01 and ***P < 0.001. Note: Right ventricular fractional area change (RV FAC%), pulmonary artery acceleration time (PAAT), and tricuspid annular plane systolic excursion (TAPSE).

To investigate the role of YWHAZ in an experimental PAH animal model, adeno-associated virus was injected into 6-week-old SD rats to construct an animal model of YWHAZ inhibition, and an AAV empty vector was used as a control injection. These rats were subjected to hypoxia and combined weekly su5416 injection interventions starting at 8 weeks of age for 4 weeks (Fig. 6A). WB results showed that adeno-associated virus injection (shYWHAZ) suppressed YWHAZ expression (Fig. 6B). Silencing of YWHAZ significantly ameliorated RVSP in PAH rats (Fig. 6C). Similarly, in echocardiographic assays, silencing YWHAZ reduced right ventricular internal diameter (RVID) and improved right ventricular fractional area change (RV FAC%), pulmonary artery acceleration time (PAAT), and tricuspid annular plane systolic excursion (TAPSE) in PAH rats (Fig. 6D). These findings indicate that YWHAZ silencing exerts beneficial effects on pulmonary hemodynamics in hypoxic PAH rats. Subsequently, we determined the effect of YWHAZ silencing on pulmonary vascular remodeling in hypoxic PAH rats (Fig. 6E). H&E staining demonstrated that YWHAZ inhibition attenuated pulmonary arterial wall thickening and alleviated luminal narrowing compared with the PAH model group. Masson staining further revealed that YWHAZ silencing significantly reduced abnormal collagen fiber deposition around pulmonary vessels. Consistently, the area of α-SMA-positive expression around pulmonary vessels was significantly reduced in the YWHAZ-silenced group, indicating that the degree of vascular muscularization was effectively alleviated. In alignment with in vitro experiments, qPCR and WB results confirmed that YWHAZ inhibition downregulated HIF1α expression in vivo (Fig. 6F, G). Additionally, YWHAZ silencing suppressed LDHA expression levels (Fig. 6H, I).

### DMOG partially reversed the ameliorative effects of YWHAZ silencing on RVSP, right heart function and pulmonary artery remodeling in PAH rats

**Fig. 7:**
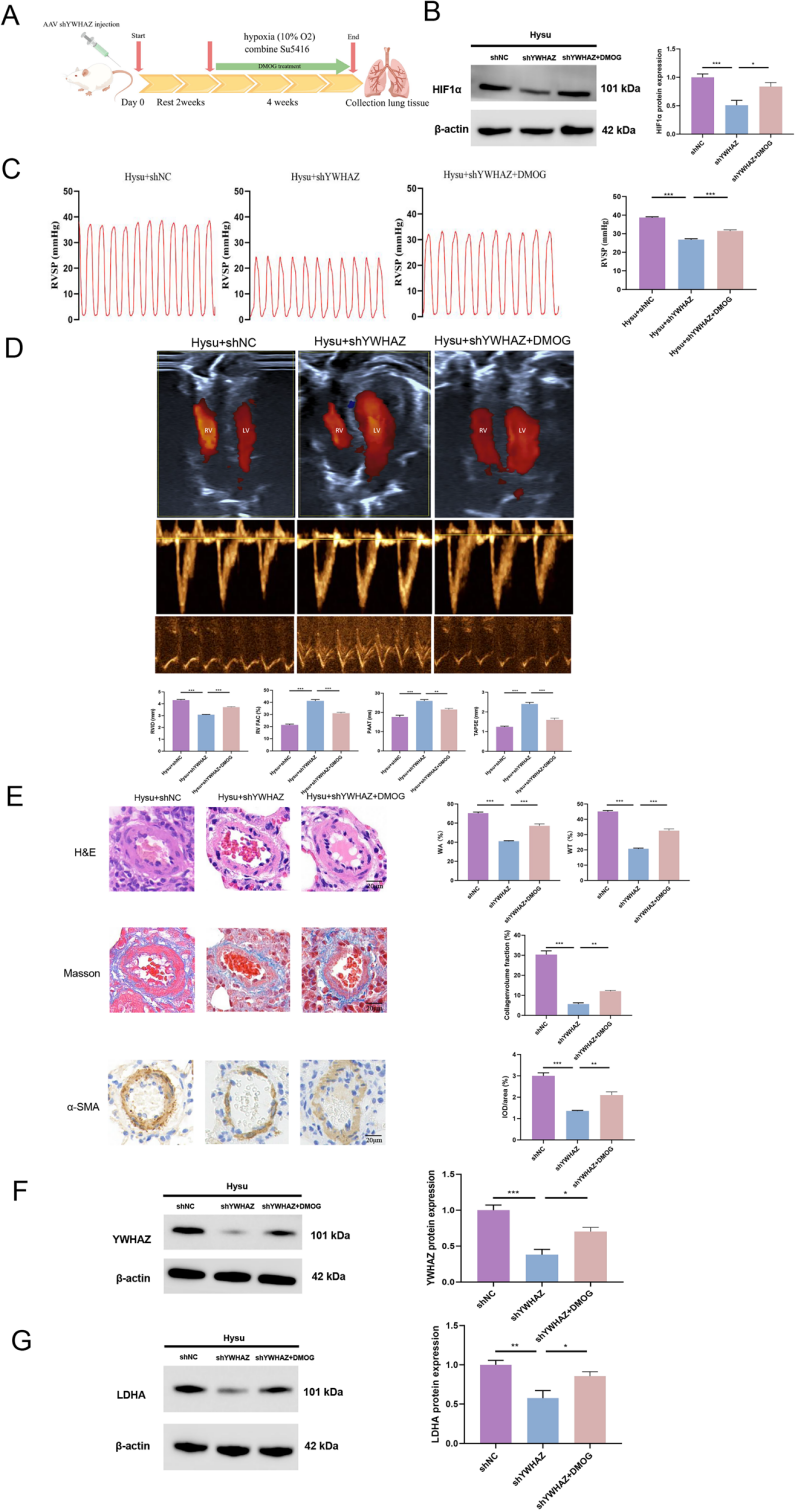
DMOG partially reversed the ameliorative effects of YWHAZ silencing on RVSP, right ventricular function, and pulmonary vascular remodeling in PAH rats. **A** Diagram of the experimental design for intervention in rats. **B** DMOG increased the expression of HIF1α in rat lung tissue (n = 6). **C** DMOG partially reversed the ameliorative effect of YWHAZ silencing on RVSP in PAH rats (n = 5). **D** DMOG partially reversed the ameliorative effects of YWHAZ silencing on RVID, RV FAC%, PAAT and TAPSE in PAH rats (n = 5). **E** DMOG partially attenuated the beneficial effects of YWHAZ silencing on pulmonary arterial remodeling, collagen deposition, and vascular muscularization (n = 6). **F**, **G** DMOG partially rescued the YWHAZ knockdown-mediated suppression of both YWHAZ and LDHA expression in lung tissues (n = 5-6). All data were presented as the mean ± SEM.*P < 0.05, **P < 0.01 and ***P < 0.001. Note: Right ventricular fractional area change (RV FAC%), pulmonary artery acceleration time (PAAT), and tricuspid annular plane systolic excursion (TAPSE).

To further validate the mechanism of YWHAZ in vivo by regulating HIF1α, we performed DMOG intervention in rats as shown in Fig. 7A. DMOG treatment increased HIF1α expression in the lung tissues of PAH rats (Fig. 7B). DMOG partially reversed the ameliorative effects of YWHAZ silencing on RVSP in PAH rats (Fig. 7C). Additionally, DMOG partially reversed the ameliorative effects of YWHAZ silencing on RVID, RV FAC%, PAAT, and TAPSE in PAH rats (Fig. 7D). Furthermore, DMOG partially reversed the ameliorative effects of YWHAZ silencing on pulmonary artery remodeling, collagen deposition, and vascular muscularization in PAH rats (Fig. 7E). The WB results demonstrated that DMOG partially reversed the inhibitory effects of YWHAZ silencing on YWHAZ expression in PAH rats (Fig. 7F). Similarly, DMOG also partially reversed the inhibitory effect of YWHAZ silencing on LDHA expression in PAH rats (Fig. 7G).

### HIF1α directly transcriptionally regulates LDHA expression

**Fig. 8:**
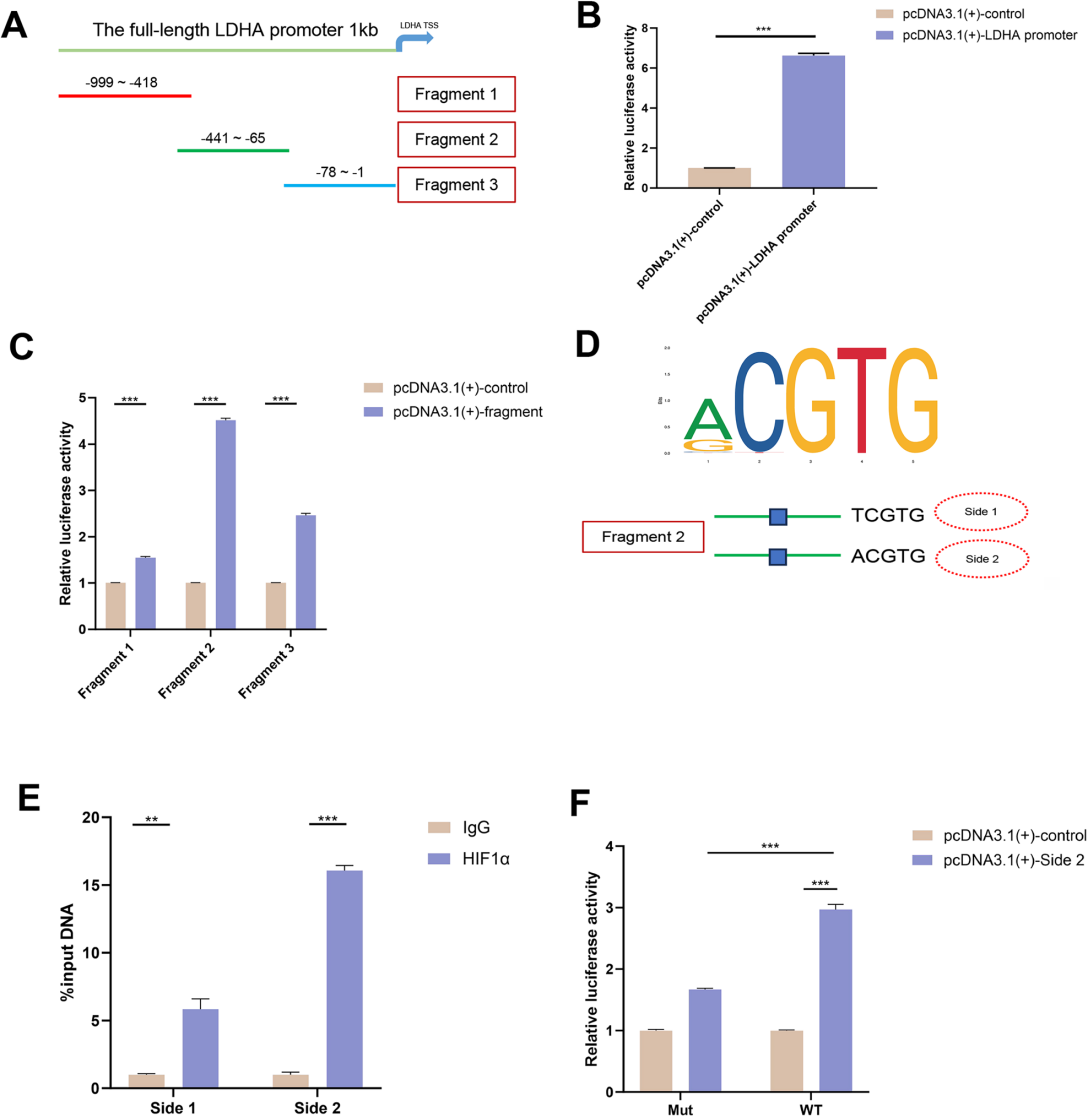
HIF1α directly transcriptionally regulated LDHA expression. **A** Schematic representation of three truncated fragments upstream of the LDHA transcription start site. **B** Dual-luciferase reporter gene assay results for the full length of the LDHA promoter (n = 3). **C** Dual-luciferase reporter gene assay results for the different truncated fragments (n = 3). **D** Putative HIF1α binding sites predicted by JASPAR database analysis. **E** CHIP-qPCR analysis of chromatin immunoprecipitated with HIF1α antibody (sites 1 and 2) (n = 3). **F** luciferase reporter gene assay analysis of LDHA promoter side 2 fragments containing the indicated mutations (n = 3). All data were presented as the mean ± SEM.*P < 0.05, **P < 0.01 and ***P < 0.001.

To explore whether HIF1α could directly interact with the LDHA gene promoter element, we constructed the LDHA promoter (1 kb) into a luciferase reporter plasmid. To pinpoint the key regulatory regions of the LDHA promoter region, we randomly designed three overlapping truncated fragments within 1000 bp upstream of the LDHA transcription start site: fragment 1 (-999 to -418bp), fragment 2 (-441 to -65bp) and fragment 3 (-78 to -1bp) (Fig. 8A). This overlapping design may avoid false-negative results due to truncation sites disrupting potential transcription factor binding sites. Using a dual-luciferase reporter assay, we found that the full-length 1000 bp LDHA promoter significantly enhanced reporter gene expression, exhibiting approximately 6-fold higher relative luciferase activity compared to the empty vector control (Fig. 8B), confirming the presence of core transcriptional regulatory elements in this region. To identify the coregulatory region of the LDHA promoter region, we performed a systematic transcriptional activity analysis of the three truncated fragments using a dual luciferase reporter system. The results showed that all the truncated fragments exhibited a significant enhancement of transcriptional activity compared with the empty vector control, but fragment 2 appeared to have a stronger transcriptional activation ability, with a higher activity than the other two fragments. Based on this finding, we chose fragment 2 as the focus of our subsequent study to deeply analyze the key regulatory elements in this region and its mechanism of action (Fig. 8C). Bioinformatic prediction using the JASPAR database identified two potential HIF1α binding sites within Fragment 2 (Fig. 8D). Subsequently, validated HIF1α‘s specific binding to both predicted regions (Site 1 and Site 2), with Site 2 showing higher affinity (Fig. 8E). To further validate this finding, we constructed a site 2 binding site mutant (site 2-Mut) and functionally verified it by a dual luciferase reporter system. Compared to the wild-type (Site 2-WT), the mutant exhibited significantly reduced luciferase activity (Fig. 8F).

Taken together, these experimental results suggest that HIF1α is able to enhance its transcriptional activity by directly binding to specific regions upstream of the LDHA transcription start site (especially site 2), thereby regulating LDHA expression at the transcriptional level. Finally, the mechanism diagram of this study is shown in Supplementary Fig. 6. The full length uncropped original western blots could be seen in Supplemental material.

## Discussion

In this study, we systematically revealed the critical regulatory role of YWHAZ in the development of PAH and its underlying molecular mechanism. We found that YWHAZ may promote the pathological progression of PAH by activating the HIF1α signaling pathway and regulating the abnormal glycolytic metabolic process in pulmonary hypertension. Previous studies have shown that pulmonary vascular endothelial cells from PAH patients exhibit significant metabolic reprogramming characterized by a shift from oxidative phosphorylation to glycolysis [11, 24]. This metabolic switch facilitates endothelial cell proliferation and apoptosis resistance [24], while glycolysis-stimulated angiogenesis further exacerbates vascular remodeling [25]. Cao et al. substantiated that PFKFB3-mediated endothelial glycolysis plays a pivotal role in PAH pathogenesis [26]. In addition, inhibition of glycolysis by down-regulation of pyruvate kinase M2 (PKM2) protein expression effectively ameliorates endothelial cell hyperproliferation [12]. These studies provide new ideas for targeting glycolysis to treat PAH. In the present study, we found that silencing YWHAZ could significantly attenuate hypoxia-induced glucose uptake and utilization by PAECs, while decreasing lactate levels and inhibiting PAECs proliferation and migration. Moreover, the current study also found that YWHAZ intervention significantly ameliorated RVSP, attenuated pulmonary vascular remodeling and fibrosis in PAH rats. YWHAZ knockdown significantly suppressed expression of LDHA, a key glycolytic enzyme, in PAH rat. These results suggest that YWHAZ may play an important role in PAH by regulating the glycolytic pathway. Metabolic reprogramming is a recognized core effector of disease pathology across diverse conditions, exemplified in cancer by gut microbiota metabolites activating pathways that promote tumor progression [27]. Our findings are consistent with previous studies and further support the regulation of metabolic reprogramming as a potential strategy for the treatment of PAH.

HIF1α, as a pivotal transcriptional regulator, plays an important role in regulating biological processes such as cell proliferation, migration and apoptosis resistance [28]. Previous study found upregulated HIF1α expression in plexiform lesions, pulmonary arteries, and cardiomyocytes of PAH patients [9]. Further investigations revealed that HIF1α levels are significantly elevated in PAH and promote adverse remodeling of pulmonary arteries [29–31]. Downregulation of HIF1α expression in pulmonary tissues and vasculature effectively inhibits pulmonary vascular remodeling, thereby ameliorating PAH [32]. Interestingly, HIF1α levels were significantly positively correlated with mean pulmonary arterial pressure in patients with hypoxia-related PAH and in experimental animal models [33, 34], which also suggests that HIF1α has an important regulatory role in the pathologic process of PAH. Our Spearman correlation analysis revealed a significant positive correlation between YWHAZ and HIF1α expression, suggesting a potential functional interplay between these molecules in PAH pathology. In vivo and in vitro experiments demonstrated that YWHAZ knockdown significantly altered HIF1α expression. The biological effects mediated by YWHAZ silencing were partially reversed upon treatment with an HIF1α agonist. Collectively, our in vivo and in vitro findings demonstrate that YWHAZ likely exerts its biological functions by modulating the HIF1α signaling pathway.

In the glycolytic pathway, lactate dehydrogenase (LDH), a key enzyme composed of LDHA or LDHB subunits [35], plays an important role in cellular metabolism. A recent study found that LDHA expression was significantly elevated in both lung tissue and heart in the monocrotaline-induced PAH rat model [36]. Wang et al. further confirmed that LDHA deficiency effectively suppresses hypoxia-induced pulmonary vascular remodeling in mice PAH models [37]. Other study further found that HIF-1α robustly enhances the expression of key glycolytic genes, including ALDOC, PGK1, and LDHA, with induction levels exceeding 2-fold, whereas HIF-2α exhibits a weaker regulatory effect (<2-fold) [38]. Early work by Semenza et al. provided evidence that HIF-1 transcriptionally regulates LDHA [39], yet the precise molecular mechanisms remain to be fully elucidated. In this study, ChIP-qPCR assays demonstrated that HIF1α specifically binds to a specific region (site 2) of the LDHA promoter. Furthermore, dual-luciferase reporter assays revealed that HIF1α enhances LDHA transcriptional activity by directly binding to its promoter, thereby upregulating LDHA expression at the transcriptional level and promoting glycolytic flux. Overall, our findings position YWHAZ as a critical hub that orchestrates the pathological coupling of HIF-1α signaling with LDHA-mediated glycolytic reprogramming in PAH. This concept parallels an emerging paradigm in tumor biology, wherein targeting master regulatory nodes—such as protein neddylation—effectively disrupts the complex signaling networks that drive microenvironment remodeling [40]. This study has limitations. Our findings are based on the hypoxia/SU5416 PAH model and rat PAECs, which may not fully represent other forms of PAH or human biology. Thus, the generalizability of the YWHAZ/HIF-1α/LDHA axis requires validation in additional models (e.g., monocrotaline-induced) and human cells. Future studies addressing these points will strengthen the translational relevance of our conclusions.

## Conclusion

In summary, our findings demonstrated that YWHAZ silencing ameliorates hypoxia-induced pulmonary vascular remodeling in PAH by suppressing glycolysis through modulation of the HIF1α/LDHA axis. This study provided a potential therapeutic target for developing novel metabolism-targeted strategies in PAH treatment.

## Methods

### Animal

The study experiments used 8-week-old male Sprague Dawley (SD) rats provided by the Animal Experiment Center of Guangxi Medical University. The study was approved by the Ethics Committee of Guangxi Medical University (Ethics No. 202309015). All methods were performed in accordance with the relevant guidelines and regulations.The rats were housed in a 12-hour light/dark cycle with the ambient room temperature maintained at 20 °C to 25 °C and humidity at 50% to 60%. The rats were provided with appropriate amounts of autoclaved feed and drinking water at regular intervals throughout the day. For the SU5416 combined hypoxia-induced PAH model (Hysu), SU5416 was dissolved in DMSO, and SU5416 (HY-10374, MCE, China) at 20 mg/kg was injected subcutaneously into the experimental rats once a week for 3 weeks, whereas the control rats received the same volume of DMSO subcutaneously. The experimental rats were exposed to chronic hypoxia (10% O₂) for 4 weeks, while control rats were maintained under normoxia for 4 weeks. After a 2-week acclimatization period, rats were intravenously injected via the tail vein with either an adeno-associated virus carrying YWHAZ-silencing sequences (shYWHAZ) or a negative control virus (shNC) to modulate pulmonary arterial YWHAZ expression. In order to investigate the effect of YWHAZ on the hypoxic PAH rat model, using a simple randomization procedure (coin toss), SD rats were randomly divided into: (1) Control group, (2) Hysu group, (3) Hysu + shNC group, and (4) Hysu + shYWHAZ group. Next, in order to investigate the effect of YWHAZ-regulated HIF1α on the hypoxic PAH rat model, SD rats were randomly divided into (1) Hysu + shNC, (2) Hysu + shYWHAZ, and (3) Hysu + shYWHAZ + DMOG (intraperitoneally injected at 40 mg/kg every other day) groups. DMOG is an agonist of HIF1α.

### Echocardiography measurement

Rats were anesthetized using 4% isoflurane, and high-frequency ultrasound (HP 7500, PHILIPS, USA) was used to detect and record right ventricular transverse diameter (RVID), tricuspid valve systolic displacement (TAPSE), right ventricular fractional area change (RV FAC%), and pulmonary artery acceleration time (PAAT). Specifically, under 2D ultrasound guidance, the ultrasound sampling line placed the sampling point in the anterior tricuspid annulus, and the M-mode ultrasound measurement of the tricuspid orifice measured the longitudinal offset of the tricuspid annulus from end-diastole to end-systole, i.e., the TAPSE. The right ventricular end-diastolic and end-systolic areas were obtained by means of 2D echocardiography and the fraction of change in area of the right ventricle, RV FAC%, was calculated. Finally, the sampled volume was placed at the pulmonary valve orifice under the guidance of 2D ultrasound to obtain the spectrum of antegrade pulmonary valve flow, and the time interval from the onset of systolic ejection to peak flow was measured in response to pulmonary arterial hypertension (a shortened PAAT is usually indicative of pulmonary arterial hypertension).

### Measurement of right ventricular systolic pressure (RVSP)

After the rats were anesthetized via isoflurane, the thoracic cavity was carefully opened and the right ventricle was accessed by gentle puncture using a 25 G needle (Terumo, #NN-2516R), followed by slow delivery of the catheter along the puncture point into the right ventricle [41]. After securing the catheter, the pressure within the right ventricle was recorded as right ventricular systolic pressure (RVSP).

### Histopathological analysis

Fixed lung tissues were paraffin-embedded and sectioned at 5 μm, then stained with hematoxylin (H8070, Solarbio, China) and eosin (A600190, Sangon, China). Pulmonary arteries with an external diameter of 25 ~ 100 µm were analyzed morphometrically and the relative thickness area was quantified using ImageJ software to calculate the ratio of pulmonary artery midlayer thickness to vessel external diameter (WT%) and the ratio of vessel midlayer area to total area (WA%). Masson staining of lung tissue was performed to assess the degree of fibrosis (collagen fiber areas stained blue). For immunohistochemistry, sections were subjected to antigenic repair to eliminate endogenous peroxidase and blocked with goat serum solution. Incubation with anti-α-SMA antibody at 4^◦^ C was performed overnight. This was followed by incubation with biotin-coupled secondary antibody (1:200 dilution, Abcam) for 30 min at 37 °C. Then diaminobenzidine was added until the reaction was terminated. Finally, slides were counterstained with hematoxylin, dehydrated, vitrified, mounted, and then observed with a light microscope.

### Culture and intervention of primary PAECs

Pulmonary arteries were rapidly dissected from rat lung tissue. The fibrous connective tissue of the outer membrane was gently removed, and the pulmonary arteries were cut into tissue blocks of approximately 1 mm^2^ and spread evenly in 25 cm^2^ culture flasks. Tissues were cultured with RPMI-1640 medium containing 20% fetal bovine serum and 1% penicillin-streptomycin at 37 °C in a 5% CO^2^ incubator, and the flasks were inverted for 2 hours and then turned over. The medium was changed every 2 days, and cell growth was observed under an inverted microscope after 4-5 days of culture. Subsequent experiments were performed using cells in logarithmic growth phase. The third generation cells were identified by immunofluorescence staining with CD31 antibody. The purity of PAECs reached more than 95% (Supplementary Figure 1). All experiments were performed on PAECs with 70-90% cell fusion from generation 3 to 5. PAECs were transfected with siYWHAZ in RPMI-1640 medium containing 10% fetal bovine serum for 48 hours. PAECs were incubated with a gas mixture containing 3% O^2^, 5% CO^2^ and 92% N^2^ under hypoxic conditions. The siRNA or AAV intervention sequences could be seen in Supplementary Table S1.

### qPCR analysis

Total RNA was extracted with Trizol. cDNA synthesis was performed using a PrimeScript RT reagent kit (Takara, Japan). Quantitative PCR (qPCR) was carried out in a Bio-Rad CFX96 Real-Time PCR Detection System using SYBR Green Master Mix (Yeasen, China). Relative mRNA expression was calculated using the 2^−ΔΔCt^ method, with β-actin as the internal control. The β-actin internal reference was used. All primer sequences are provided in Supplementary Table S2.

### Western blot

Proteins were extracted from lung tissue or cells using RIPA with protease inhibitor (PMSF). Proteins were denatured by adding loading buffer and boiling. These proteins were then separated by 10% Express Cast PAGE (NCM Biotech, China), transferred to Millipore’s PVDF membranes (Boston, USA), and blocked with 5% skim milk. The PVDF membranes were incubated overnight at 4 °C with specific primary antibodies: HIF1α (1:1000, ThermoFisher, USA), YWHAZ (1:5000, Abmart, China), LDHA (1:1000, Proteintech, China), anti-Ubiquitin(1:1000, Proteintech, China) and β-actin ((1:5000, Proteintech, China). All blots were visualized using the e-BLOT imaging system (e-BLOT, China) and quantified using Image J (NIH, Bethesda, USA) with β-actin as a control.

### Glucose uptake and Lactate production measurement

Glucose uptake and lactate production in cell supernatants were measured by Glucose Assay (F006-1-1, Jiancheng Bioengineering, China), and Lactate Assay Kit (A019, Jiancheng Bioengineering, China) according to the manufacturer’s instructions.

### Immunofluorescence

After antigen repair of the sections, endogenous peroxidase was eliminated using 3% H_2_O_2_. Next, antigens were blocked with 5% bovine serum albumin to block nonspecific antigens. Follow-up was performed according to the manufacturer’s instructions (RS0035, Immunoway). Immunofluorescence staining was performed using rabbit anti-YWHAZ, rabbit anti-HIF1a, and rabbit anti-CD31 antibodies. Microscopic images were performed using a fluorescence microscope (Panoramic Scan II, 3DHISTECH Ltd.).

### RNA sequencing

Total RNA was extracted with Trizol reagent according to standard methods. After detection and quality control, RNA sequencing was performed on the MGI sequencing platform library (BGI, China). Differentially expressed genes (DEGs) were identified using the limma package (adjust *P* < 0.05 and |Log2FC | >1). Gene set enrichment analysis (GSEA) was then performed on the sequencing data and the results were visualized.

### EDU assay

Proliferating cells were labeled using the BeyoClick™ EdU-594 Imaging Kit (Beyotime, China) according to the instructions. Edu admixture rate was expressed as the ratio of edu-positive cells to total DAPI-positive cells.

### Transwell assay

Following treatment, PAECs were trypsinized, resuspended in serum-free medium, and seeded (5×10^4^cells/well) into the upper chamber of 24-well transwell plates (8 µm pore size, Corning). The lower chamber contained 500 µL of complete medium with 20% FBS as a chemoattractant. After 24 hours of incubation, the PAECs were stabilized with 4% paraformaldehyde for 15 minutes and then stained with crystal violet solution (G1064, Solarbio, China) for 20 minutes. Images of PAECs located on the inferior side of the luminal membrane were subsequently captured by inverted microscopy and quantitatively analyzed.

### Co-Immunoprecipitation

The Co-IP assay was performed using the Pierce Co-IP Kit (#26149, Thermo Fisher Scientific, USA) according to the manufacturer’s protocol. Briefly, PAECs were lysed using lysate and cell lysates were collected. Incubate with HIF-1α or YWHAZ antibody with pierce magnetic beads Co-IP kit for 4 h. The antibody-coated magnetic beads were then added to the above cell lysates for protein precipitation. Finally western blot assay was performed for detection.

### ChIP-qPCR

The ChIP assay kit (Millipore, Bedford, MA) was used according to the manufacturer’s instructions. Crosslinked chromatin was broken into DNA fragments by sonication, and anti-HIF-1α antibody was used to chromatin precipitate the DNA fragments. Input DNA (before immunoprecipitation) and immunoprecipitated DNA samples were analyzed by real-time PCR. Primer sequences are listed in the Supplementary Table S1. The expression levels of the binding sequences were normalized to the input DNA of these sequences.

### Luciferase reporter assay

Negative control, Wild-type (WT) plasmid and different fragments of plasmids were co-transfected with firefly luciferase and renilla luciferase into 293 T cells, respectively. The medium was replaced with fresh medium after 6 h of transfection, and 3% O₂ hypoxia was given after 24 h. The luciferase activity was detected after 48 h of transfection.

### Statistical analysis

All experimental data were presented as mean ± standard error (Mean ± SEM) and performed using GraphPad Prism 10.0 and R. Comparisons between two groups were performed using two-tailed unpaired student t-test. For comparisons among three or more groups, one-way analysis of variance (ANOVA) with tukey multiple comparison correction was used to detect intergroup differences. The correlation analysis between the two variables was conducted using the Spearman test. *P* value < 0.05 was considered statistically significant.

## Supplementary information

Supplementary Figure 1

Supplementary Figure 2

Supplementary Figure 3

Supplementary Figure 4

Supplementary Figure 5

Supplementary Figure 6

supplementary information

Supplemental Material

## Data Availability

The data that support the findings of this study are available from the corresponding author upon reasonable request.
