# Supplementary figures and images for: YWHAZ-mediated metabolic reprogramming via HIF1A/LDHA signaling promotes pulmonary arterial remodelling
Source: Cell Death Discov. 2026 May 5;12:278. doi: 10.1038/s41420-026-03121-y (PMC13287781; doi:10.1038/s41420-026-03121-y)

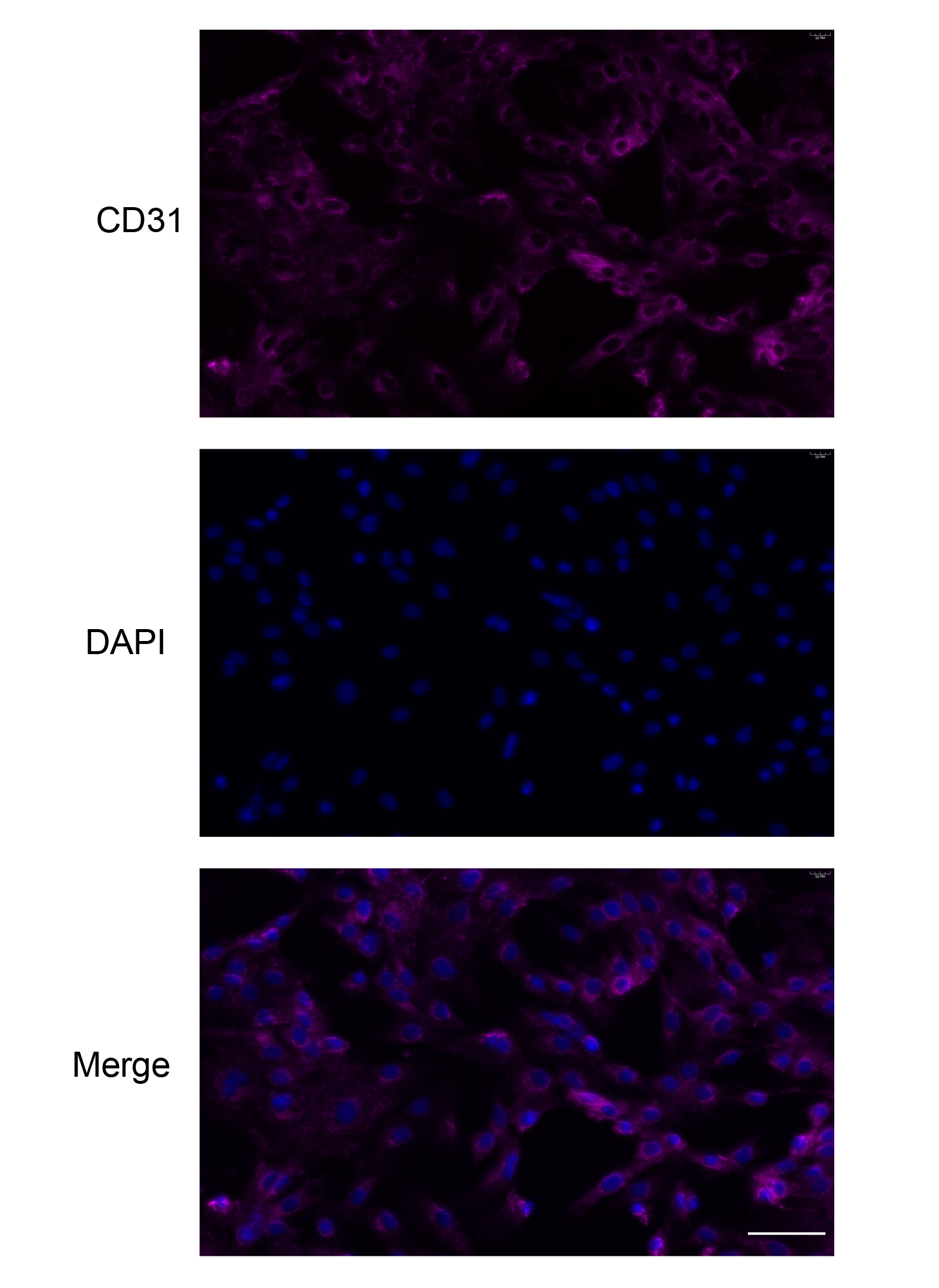

Supplement: Supplementary file 1 — Supplementary Figure 1 [file 41420_2026_3121_MOESM1_ESM.tif]

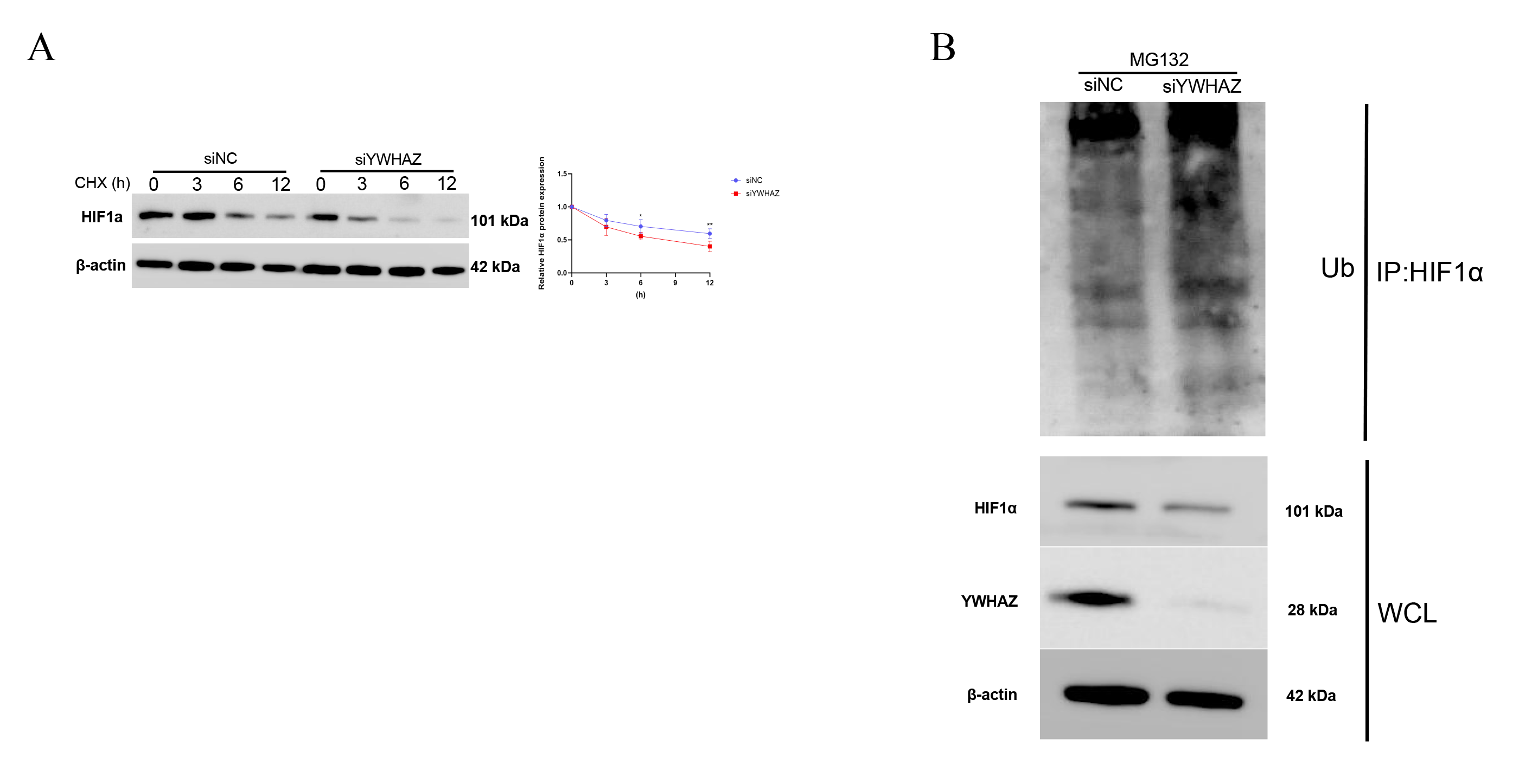

Supplement: Supplementary file 2 — Supplementary Figure 2 [file 41420_2026_3121_MOESM2_ESM.tif]

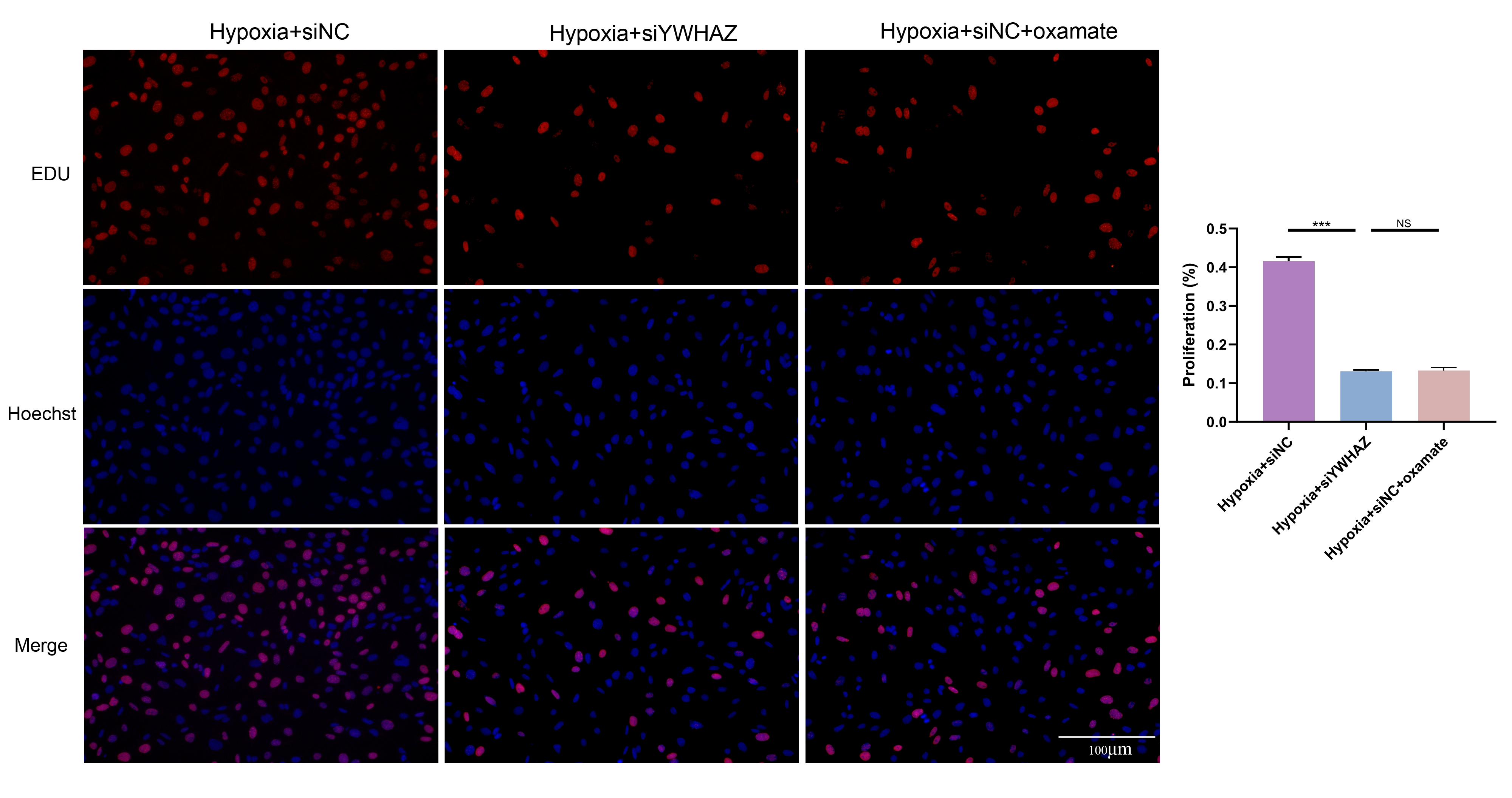

Supplement: Supplementary file 3 — Supplementary Figure 3 [file 41420_2026_3121_MOESM3_ESM.tif]

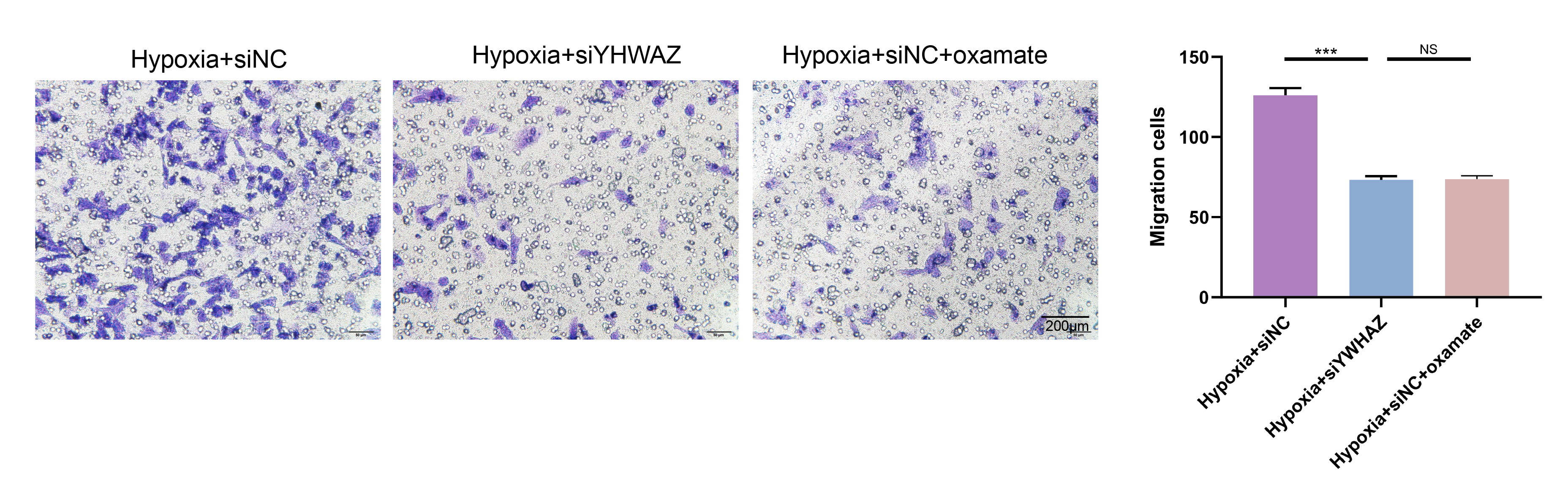

Supplement: Supplementary file 4 — Supplementary Figure 4 [file 41420_2026_3121_MOESM4_ESM.tif]

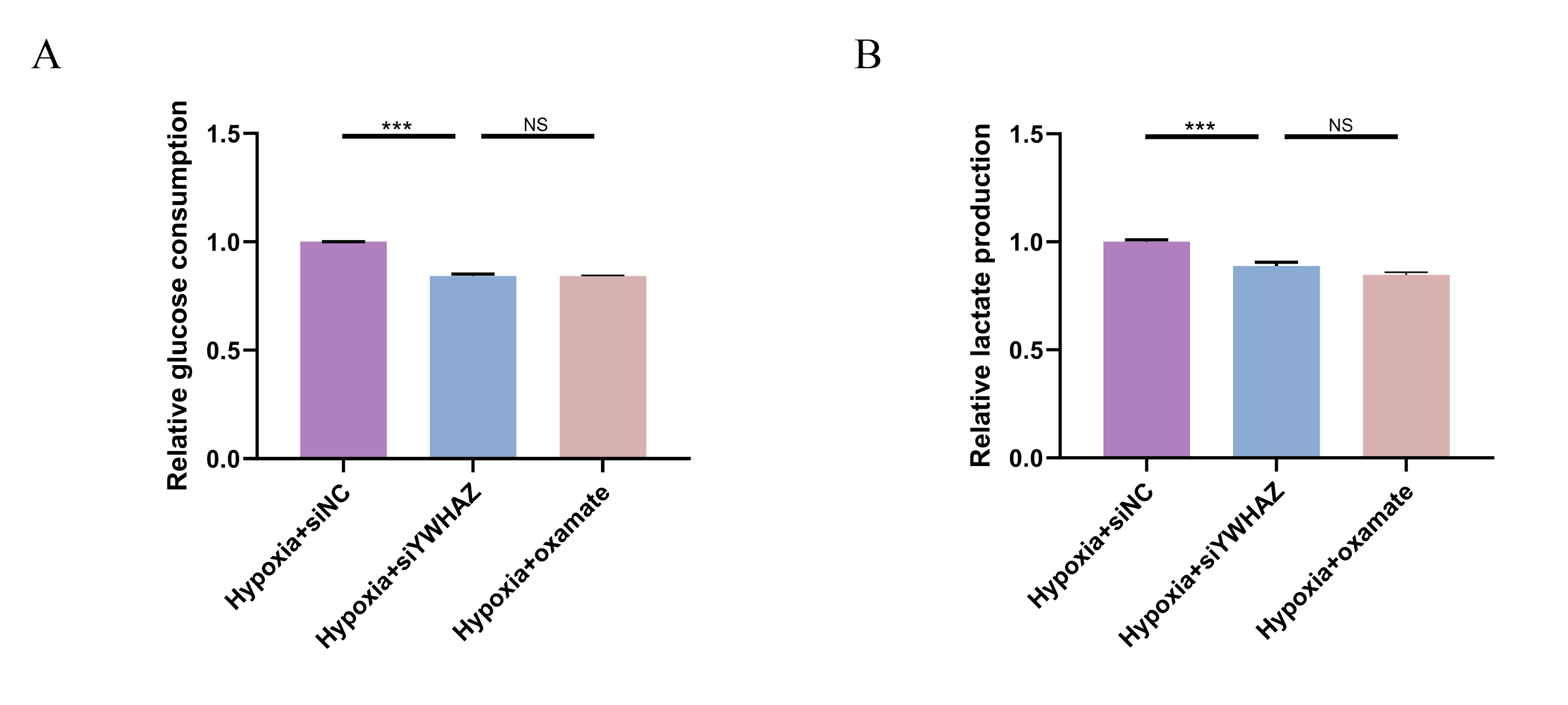

Supplement: Supplementary file 5 — Supplementary Figure 5 [file 41420_2026_3121_MOESM5_ESM.tif]

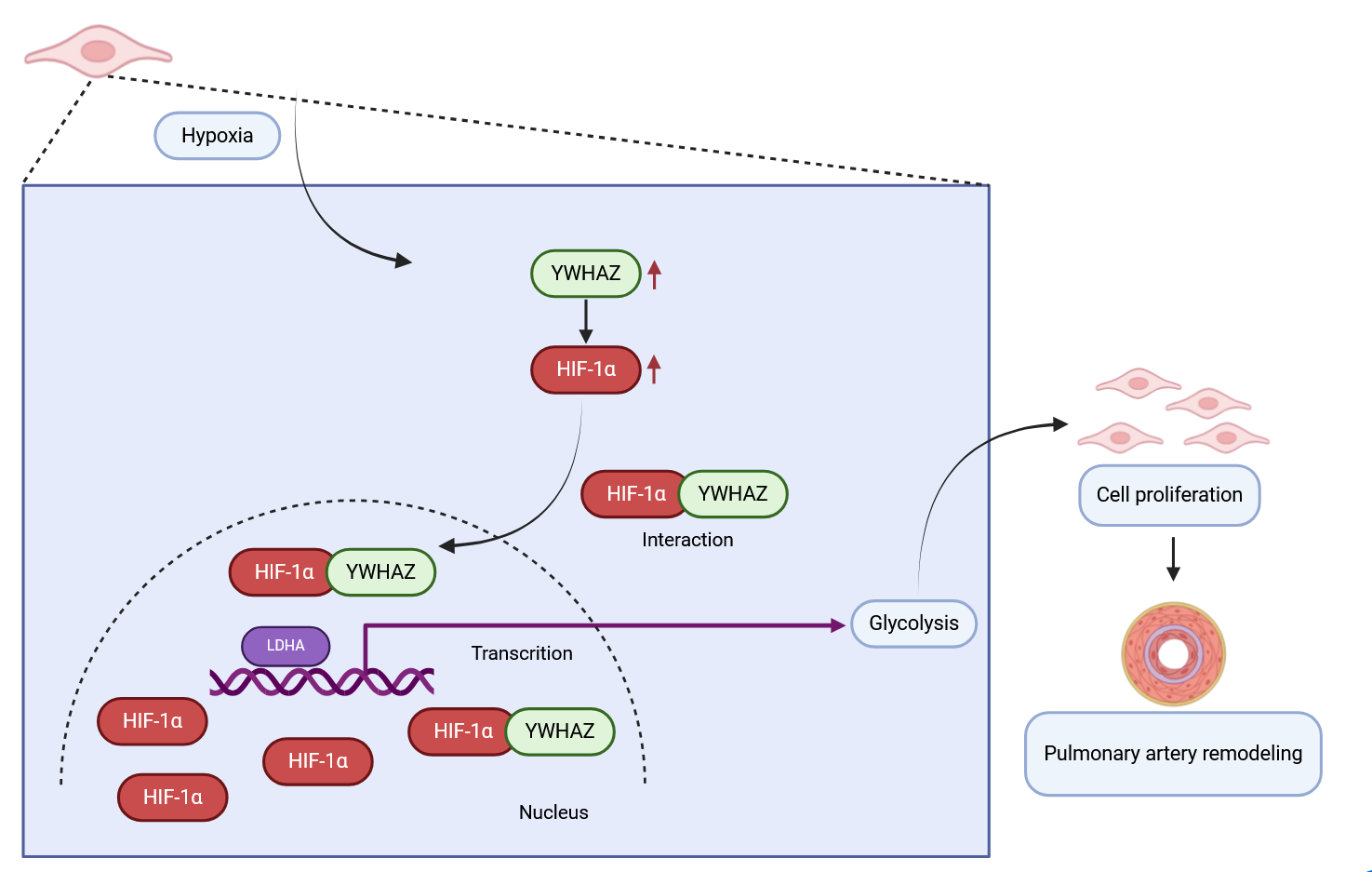

Supplement: Supplementary file 6 — Supplementary Figure 6 [file 41420_2026_3121_MOESM6_ESM.tif]
