# Supplementary material for: YWHAZ-mediated metabolic reprogramming via HIF1A/LDHA signaling promotes pulmonary arterial remodelling
Source: Cell Death Discov. 2026 May 5;12:278. doi: 10.1038/s41420-026-03121-y (PMC13287781; doi:10.1038/s41420-026-03121-y)
Supplement: Supplementary file 7 — supplementary information [file 41420_2026_3121_MOESM7_ESM.docx]

Supplementary Figure 1. Identification of primary endothelial cells by immunofluorescence.

Supplementary Figure 2.YWHAZ silencing significantly reduced HIF-1α protein stability and increased the ubiquitination of HIF-1α. (A) YWHAZ silencing significantly reduced HIF-1α protein stability; (B)YWHAZ silencing significantly enhanced ubiquitination of HIF-1α.

Supplementary Figure 3. Inhibition of LDHA and silencing of YWHAZ showed no significant difference in the proliferation.

Supplementary Figure 4. Inhibition of LDHA and silencing of YWHAZ showed no significant difference in the migration.

Supplementary Figure 5. Inhibition of LDHA and silencing of YWHAZ showed no significant difference in glucose uptake, or lactate production.

Supplementary Figure 6. The mechanism diagram of this study.

Supplementary Table S1. The siRNA or AAV intervention sequences

|  | Target sequence |
| --- | --- |
| si1-YWHAZ | F: ACUUCUCUGUGUUCUACUAUGTT  R: CAUAGUAGAACACAGAGAAGUTT |
| si2-YWHAZ | F: GAGAGAAGAUCGAGACGGAGCTT  R: GCUCCGUCUCGAUCUUCUCUCTT |
| si3-YWHAZ | F: GCAUGAAGUCUGUCACUGAGCTT  R: GCUCAGUGACAGACUUCAUGCTT |
| siNC | F: UUCUCCGAACGUGUCACGUTT  R: ACGUGACACGUUCGGAGAATT |
| AAV-shYWHAZ | GCATGAAGTCTGTCACTGAGC |
| AAV-shNC | TTCTCCGAACGTGTCACGT |

Supplementary Table S2. Primer sequences for quantitative real-time PCR.

| **Gene** | **Forward** | **Reverse** |
| --- | --- | --- |
| Rat YWHAZ | ACTACTACCGCTACTTGGCTGAGG | TTCTTGGTATGCTTGCTGTGACTGG |
| Rat HIF1α | CCGCCACCACCACTGATGAATC | GTGAGTACCACTGTATGCTGATGCC |
| Rat LDHA | CAATCTGGATTCGGCTCGGTTC | CGGCGACATTCACACCACTC |
| Rat β-actin | GGAGATTACTGCCCTGGCTCCTA | GACTCATCGTACTCCTGCTTGCTG |
| Side 1 | TACGGAGAAGGAAGCGGAGGAT | GCTAGGCGCCACCAAGATCGGG |
| Side 2 | GCCCCGATCTTGGTGGCGCCTA | TCCGTGCTGACGTCAGAGTGGA |
