# Supplementary material for: YWHAZ-mediated metabolic reprogramming via HIF1A/LDHA signaling promotes pulmonary arterial remodelling
Source: Cell Death Discov. 2026 May 5;12:278. doi: 10.1038/s41420-026-03121-y (PMC13287781; doi:10.1038/s41420-026-03121-y)

Figure 1D

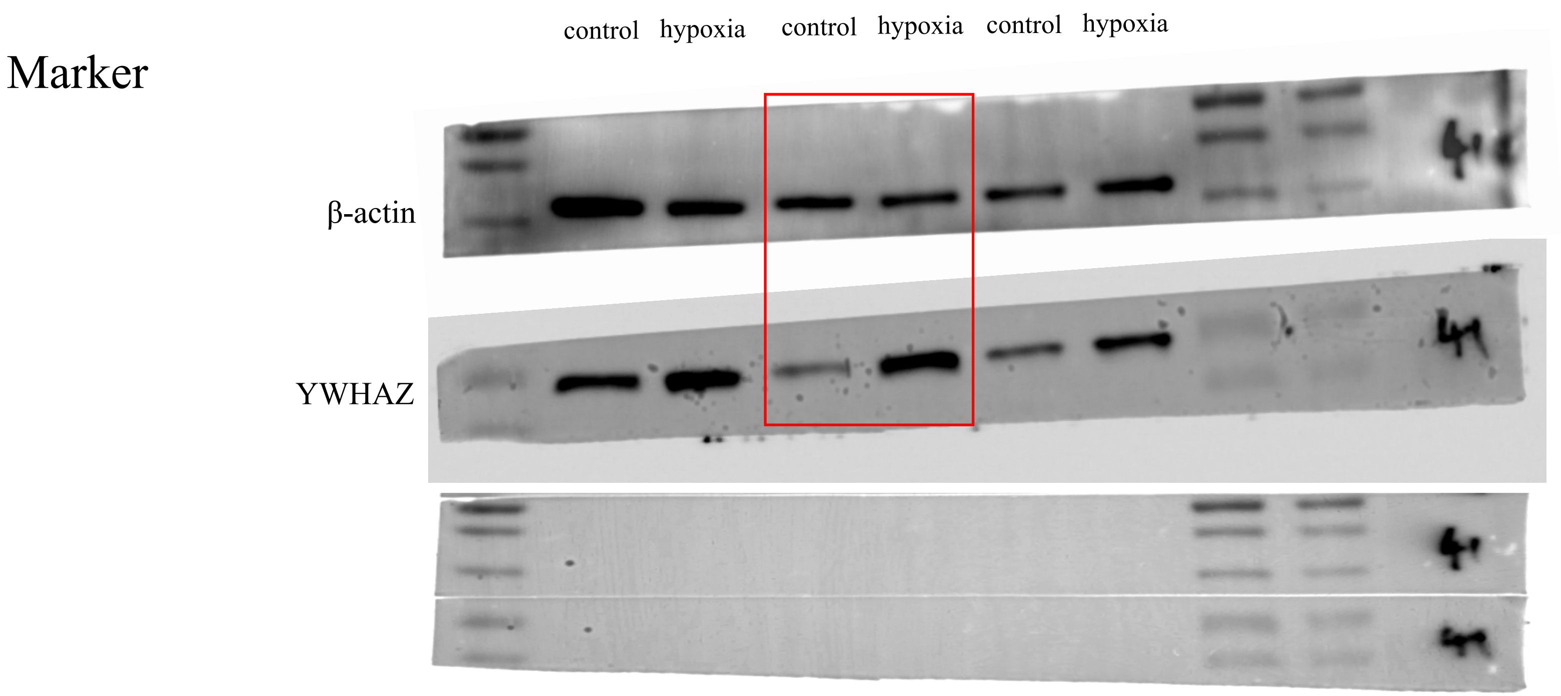

Marker
control
hypoxia
control
hypoxia
control
hypoxia
β-actin
YWHAZ

Figure 1H

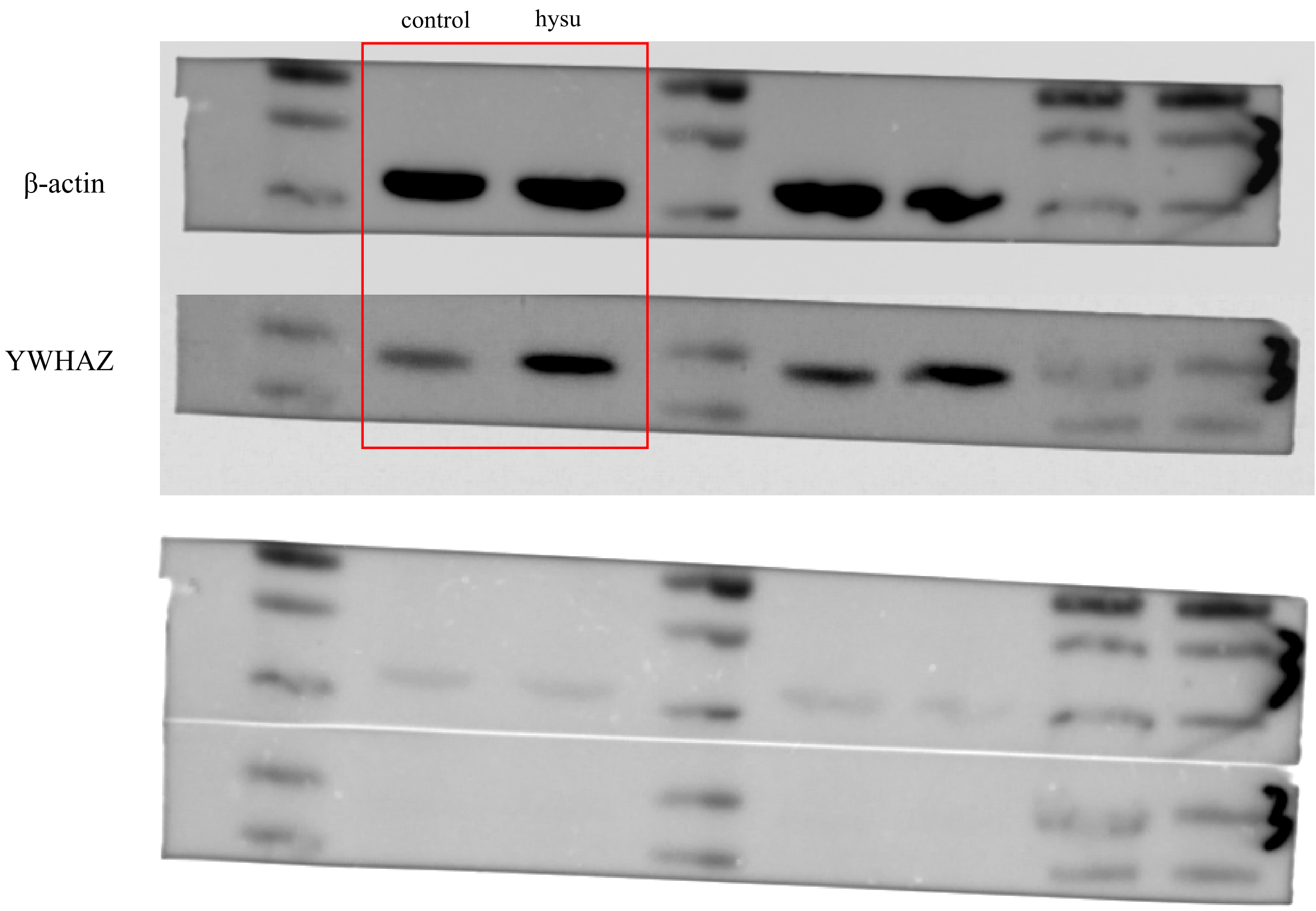

Figure 2A

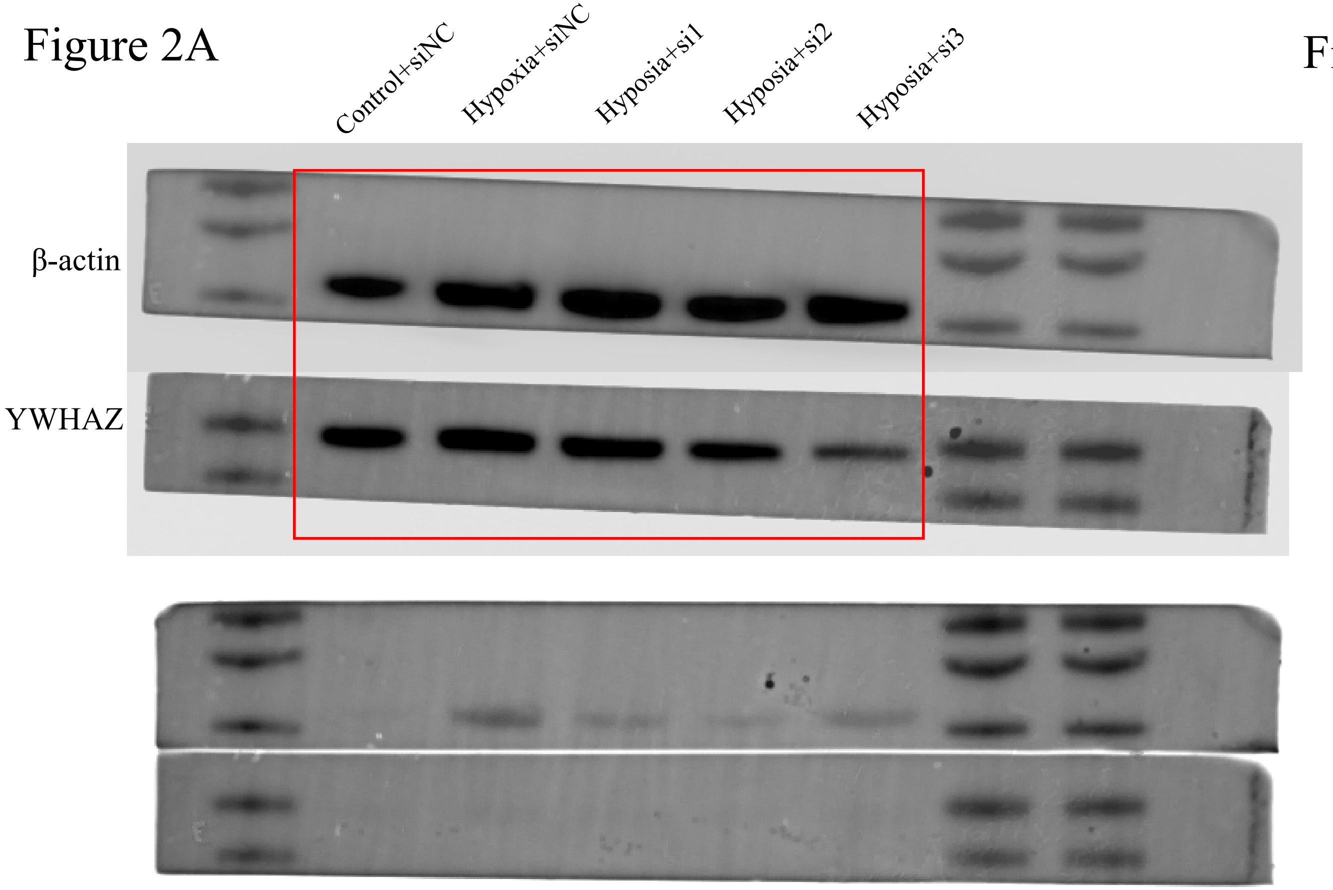

Figure 2B

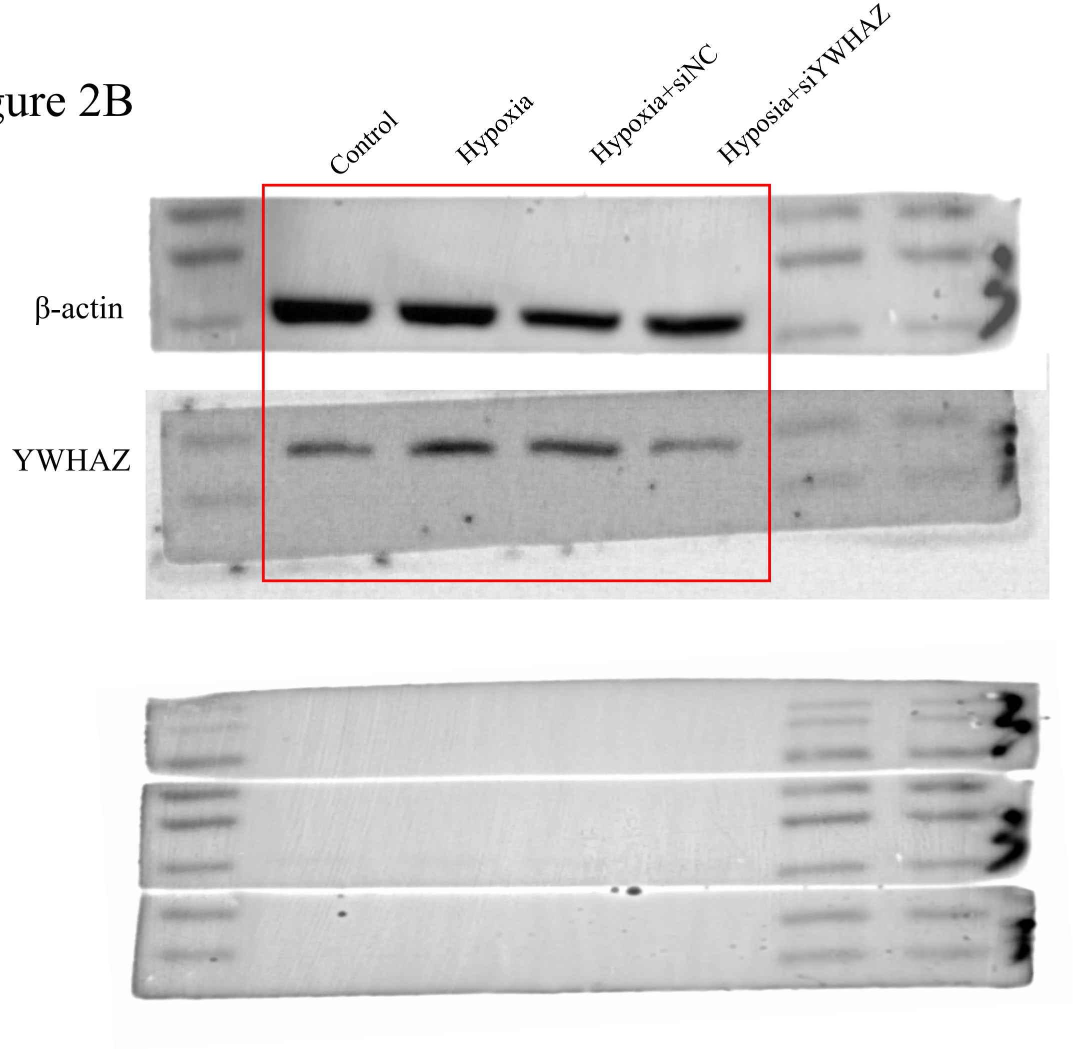

Figure 3C

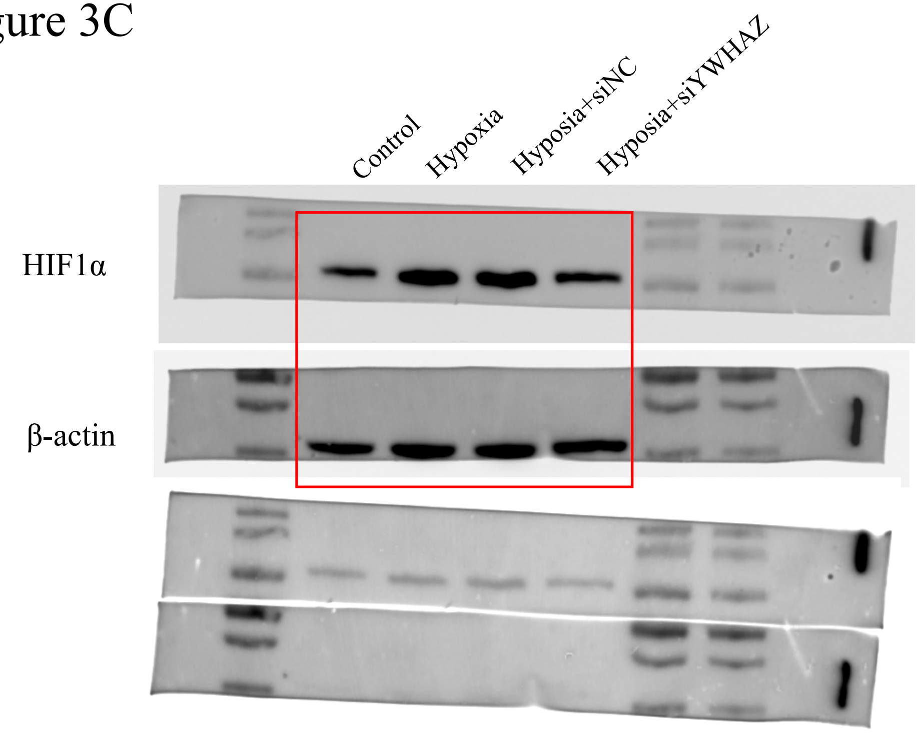

Figure 3E

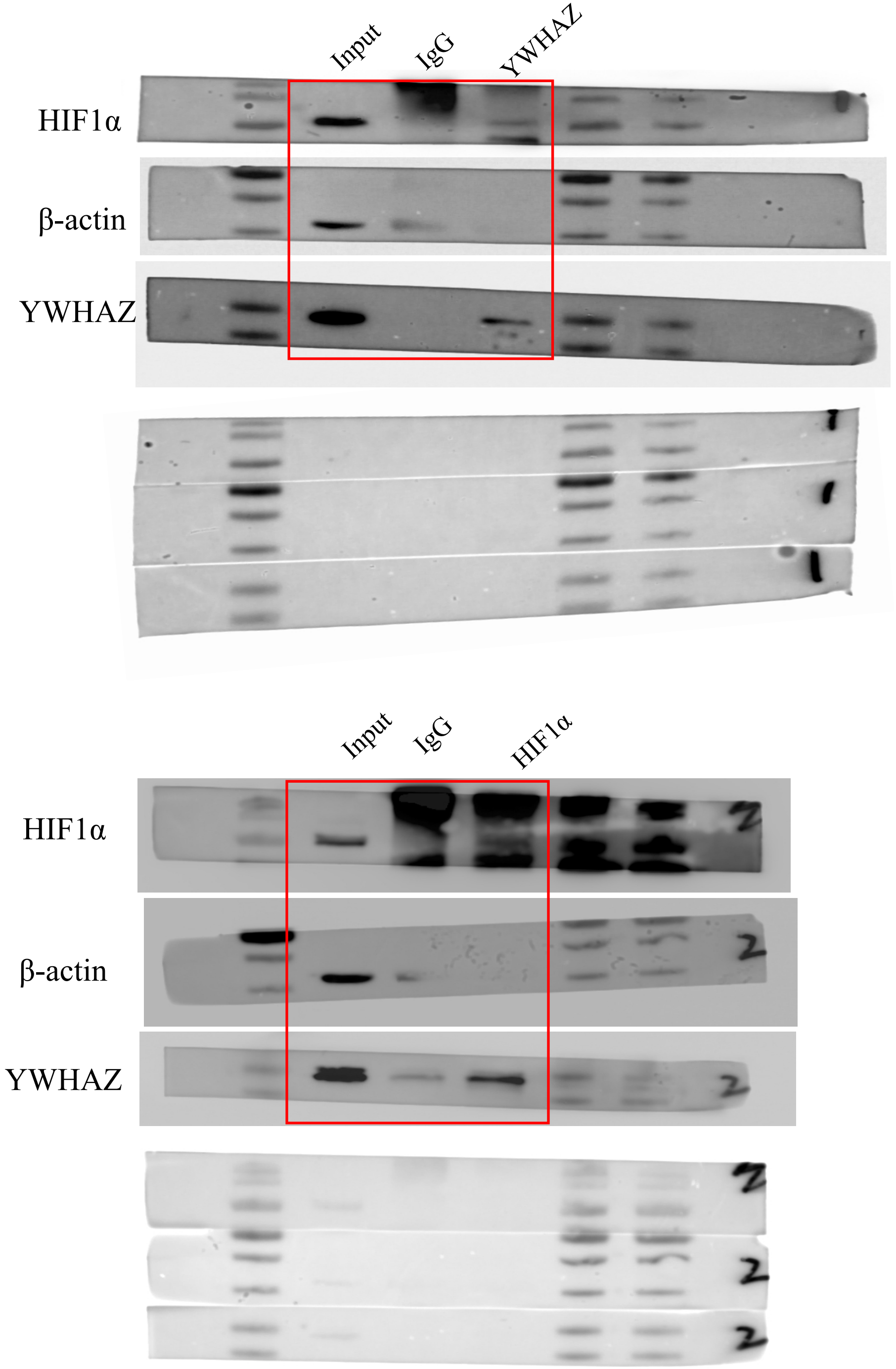

Figure 4B

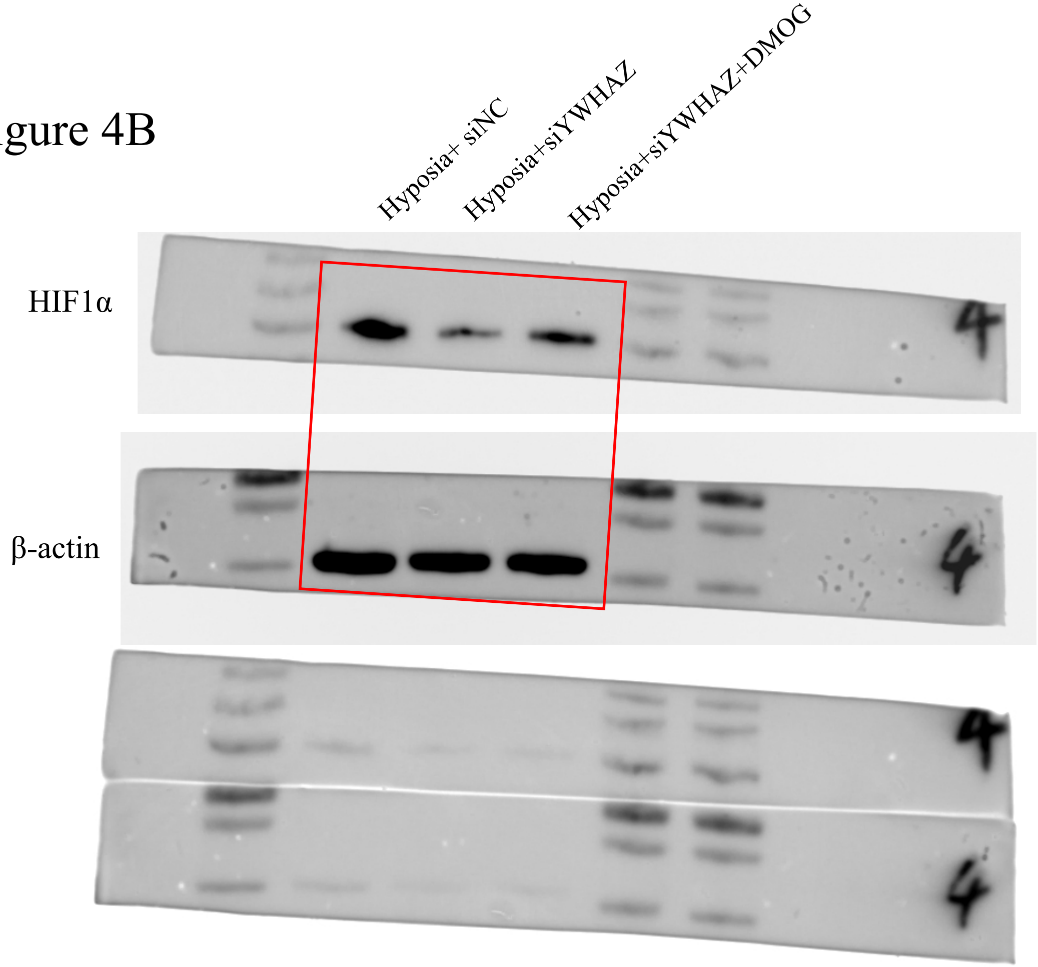

Figure 4D

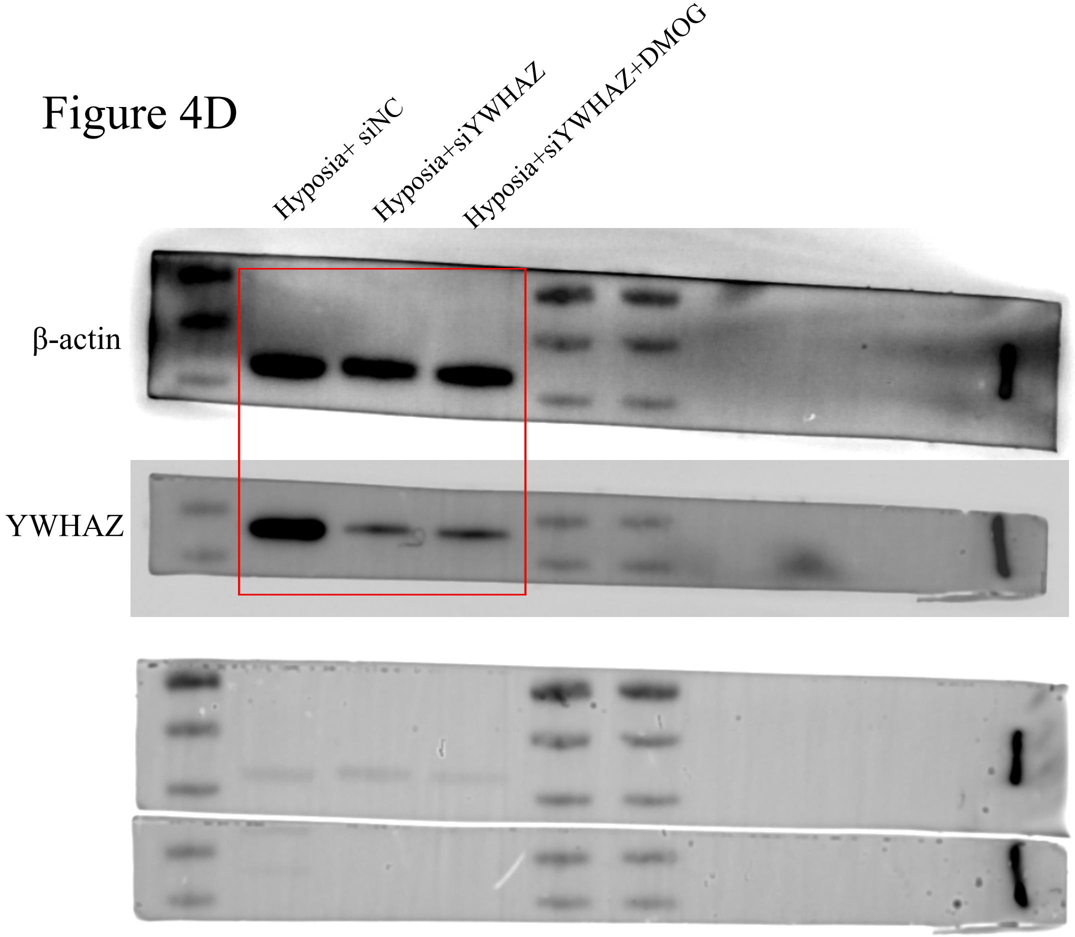

Figure 5C

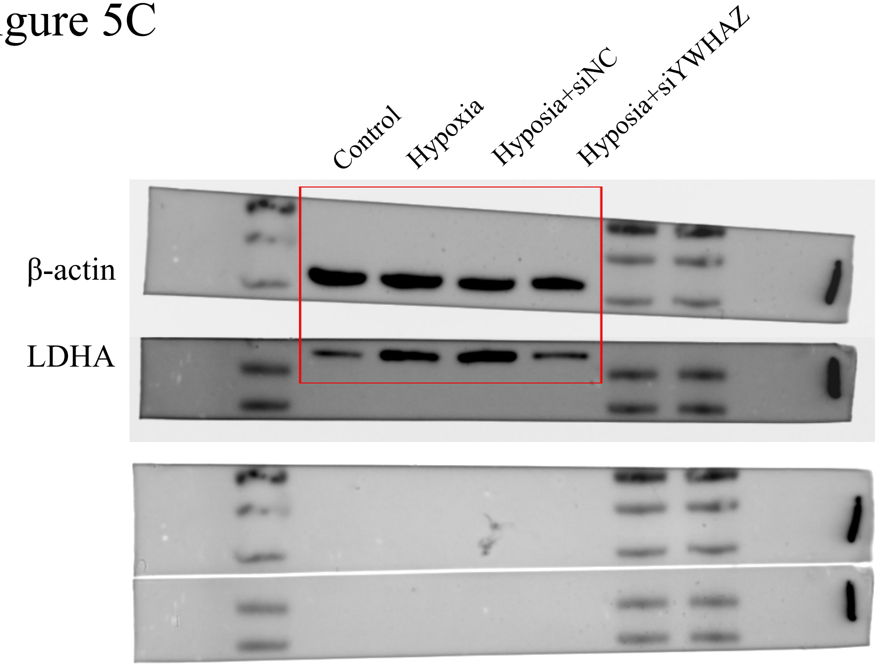

Figure 5E

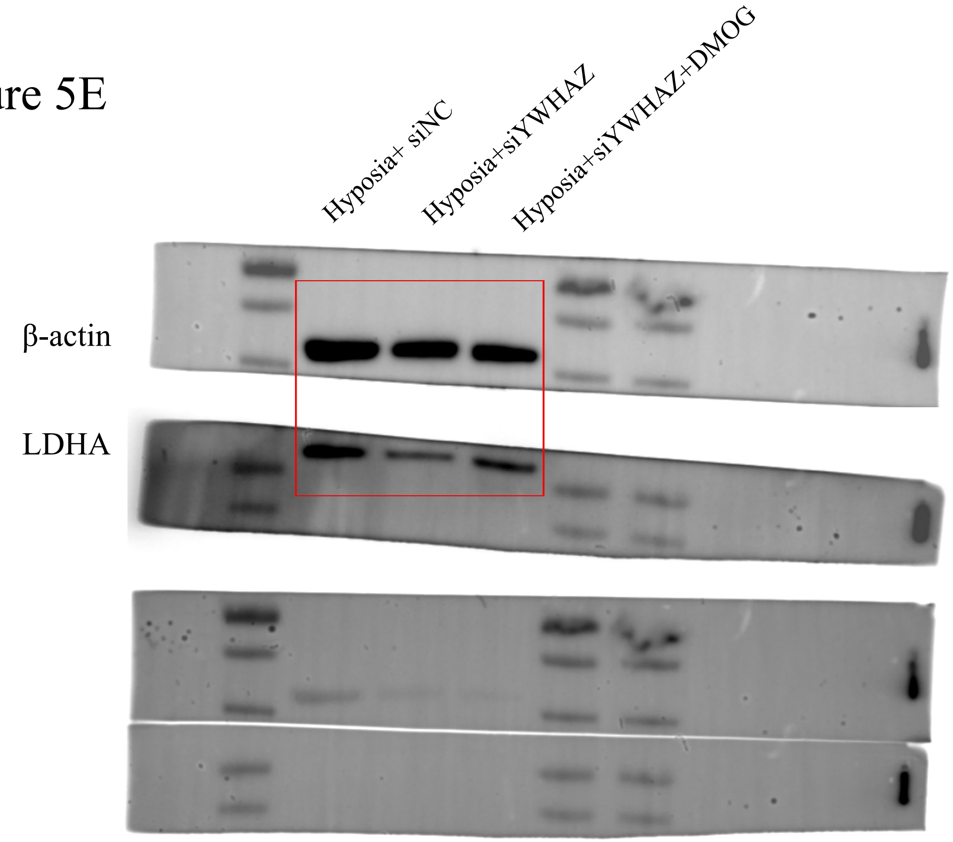

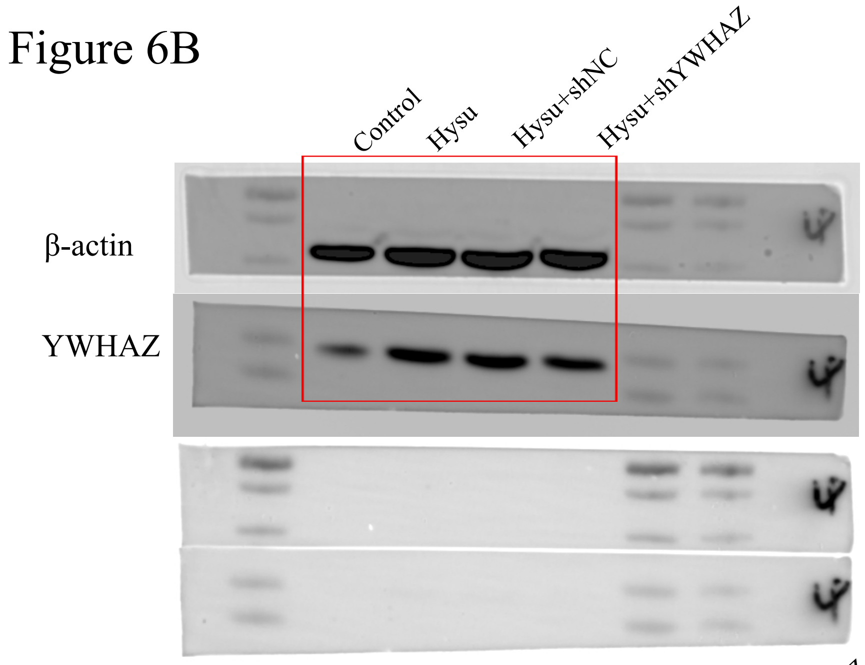
Figure 6B
Control
Hysu
Hysu+shNC
Hysu+shYWHAZ
β-actin
YWHAZ

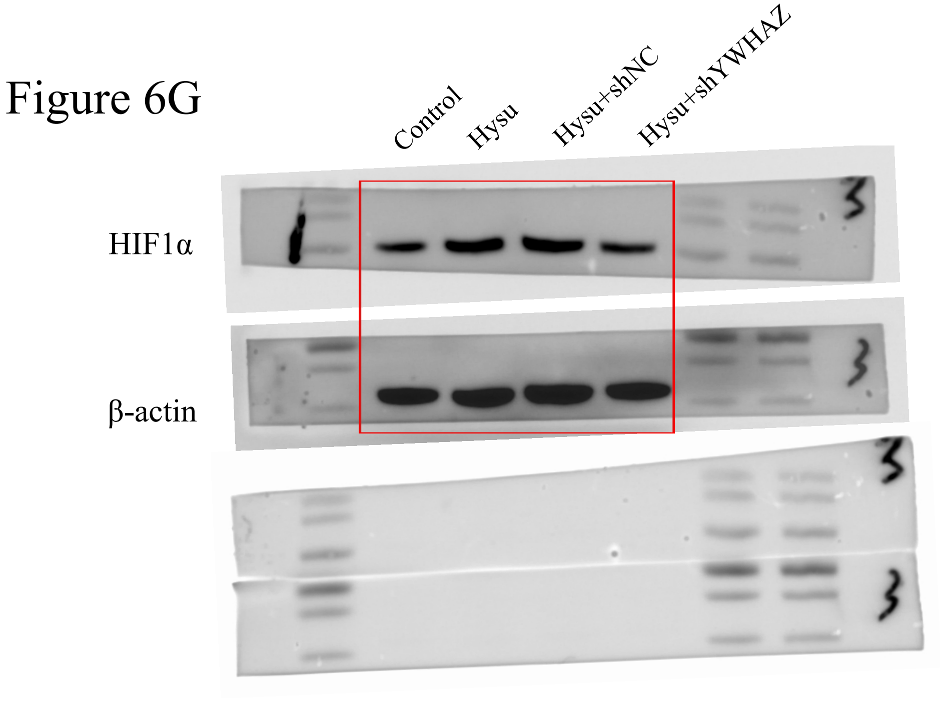
Figure 6G
Control
Hysu
Hysu+shNC
Hysu+shYWHAZ
HIF1α
β-actin

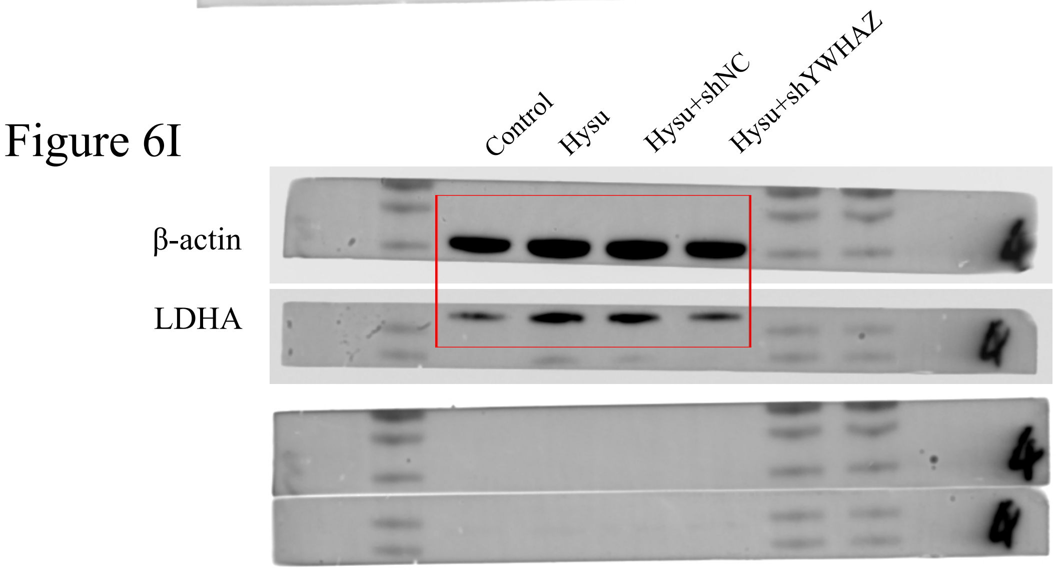
Figure 6I
Control
Hysu
Hysu+shNC
Hysu+shYWHAZ
β-actin
LDHA

Figure 7B

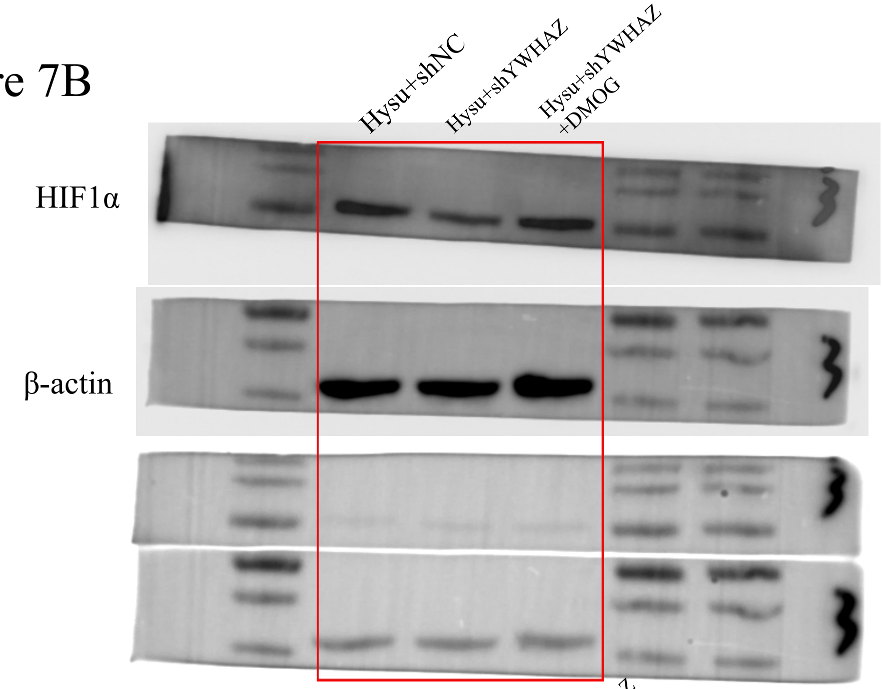

Figure 7F

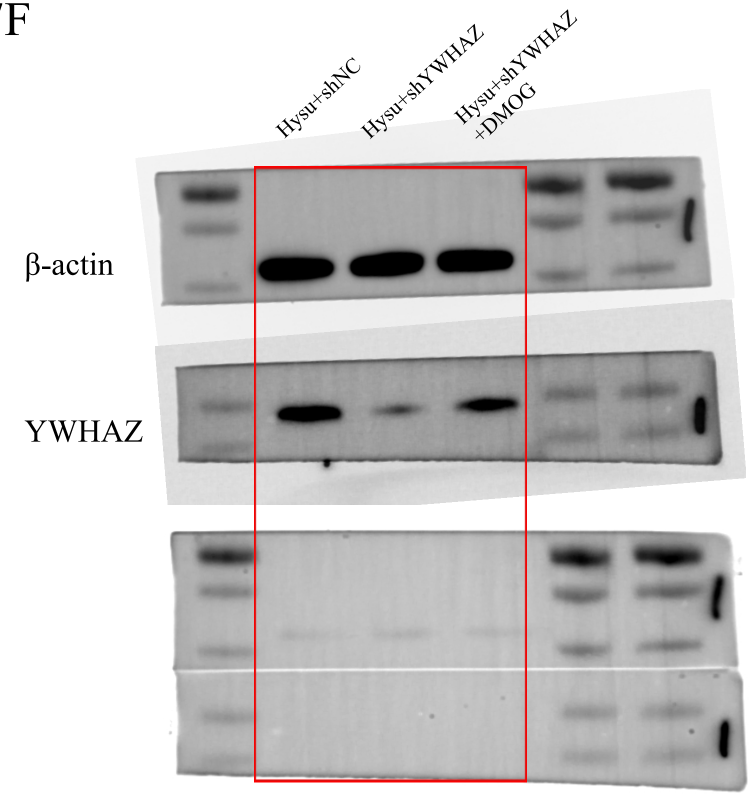

Figure 7G

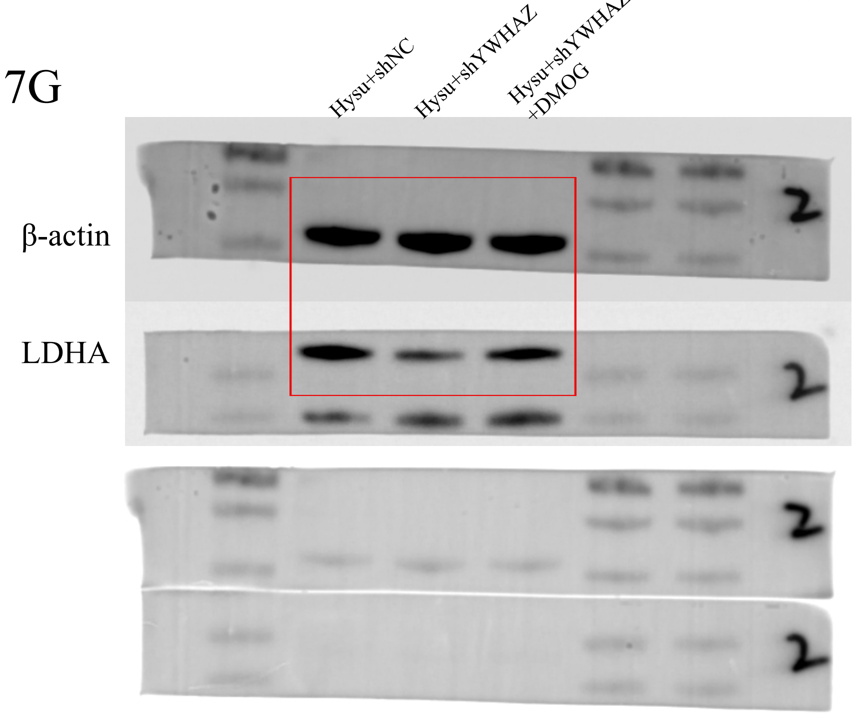

Supplementary Figure 2A

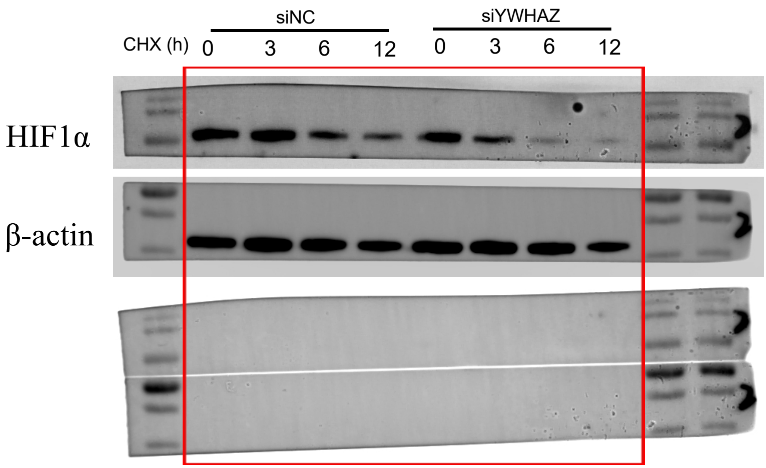

Supplementary Figure 2B

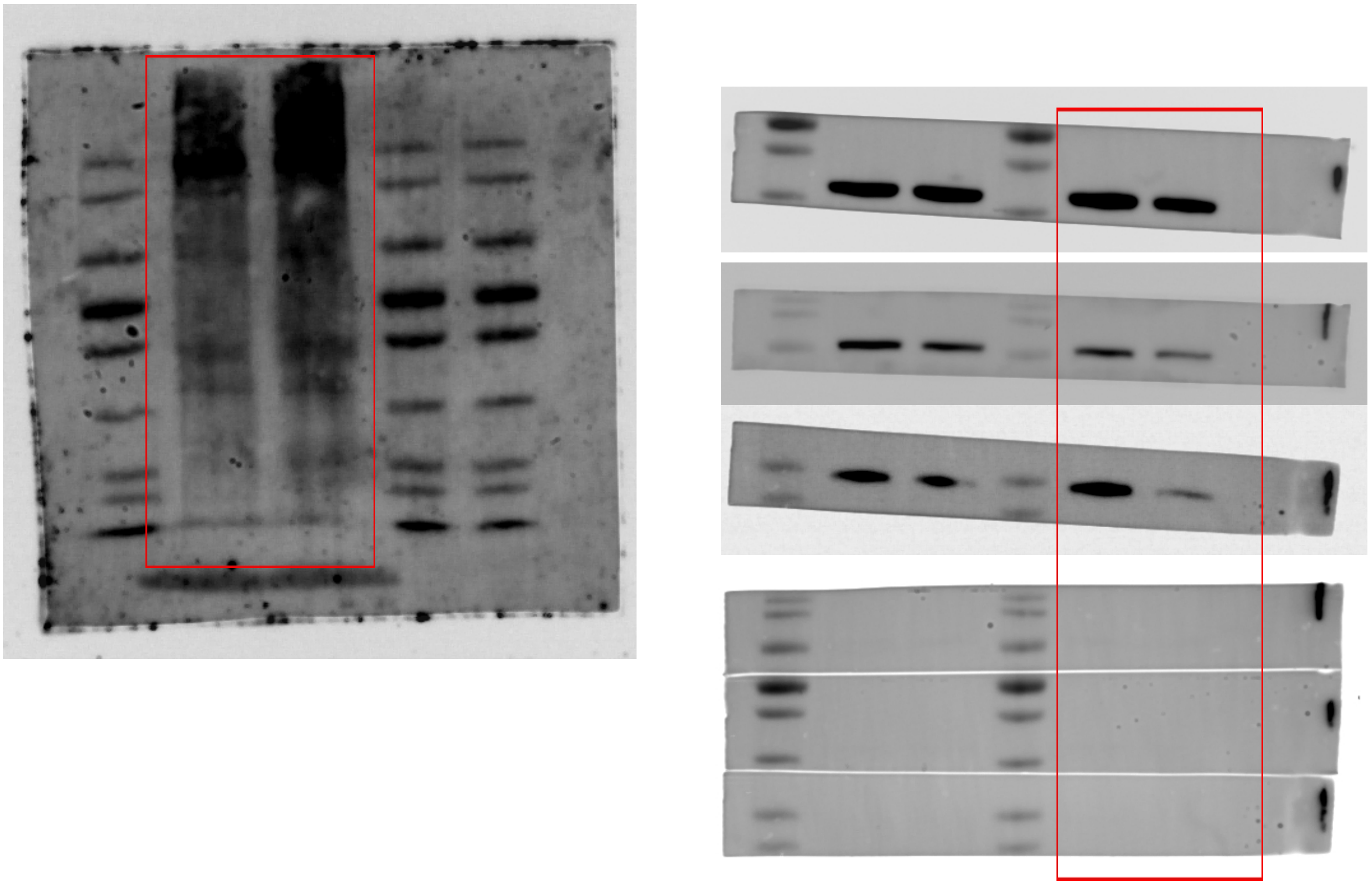

Supplement: Supplementary file 8 — Supplemental Material [file 41420_2026_3121_MOESM8_ESM.pdf]
